# Functional Assessment of Stroke-Induced Regulation of miR-20a-3p and Its Role as a Neuroprotectant

**DOI:** 10.1007/s12975-021-00945-x

**Published:** 2021-09-27

**Authors:** Taylor E. Branyan, Amutha Selvamani, Min Jung Park, Kriti E. Korula, Kelby F. Kosel, Rahul Srinivasan, Farida Sohrabji

**Affiliations:** 1 Women’s Health in Neuroscience Program, Neuroscience and Experimental Therapeutics, Texas A&M Health Science Center College of Medicine, Bryan, TX 77807 USA; 2 Texas A&M Institute for Neuroscience, College Station, TX 77840 USA; 3 Department of Neuroscience and Experimental Therapeutics, Texas A&M Health Science Center College of Medicine, 8447 Riverside Pkwy, Bryan, TX 77807 USA

**Keywords:** Ischemic stroke, MicroRNA, Astrocyte, Blood–brain barrier, Mitochondria, Matrix metalloproteinases

## Abstract

MicroRNAs have gained popularity as a potential treatment for many diseases, including stroke. This study identifies and characterizes a specific member of the miR-17–92 cluster, miR-20a-3p, as a possible stroke therapeutic. A comprehensive microRNA screening showed that miR-20a-3p was significantly upregulated in astrocytes of adult female rats, which typically have better stroke outcomes, while it was profoundly downregulated in astrocytes of middle-aged females and adult and middle-aged males, groups that typically have more severe stroke outcomes. Assays using primary human astrocytes and neurons show that miR-20a-3p treatment alters mitochondrial dynamics in both cell types. To assess whether stroke outcomes could be improved by elevating astrocytic miR-20a-3p, we created a tetracycline (Tet)-induced recombinant adeno-associated virus (rAAV) construct where miR-20a-3p was located downstream a glial fibrillary acidic protein promoter. Treatment with doxycycline induced miR-20-3p expression in astrocytes, reducing mortality and modestly improving sensory motor behavior. A second Tet-induced rAAV construct was created in which miR-20a-3p was located downstream of a neuron-specific enolase (NSE) promoter. These experiments demonstrate that neuronal expression of miR-20a-3p is vastly more neuroprotective than astrocytic expression, with animals receiving the miR-20a-3p vector showing reduced infarction and sensory motor improvement. Intravenous injections, which are a therapeutically tractable treatment route, with miR-20a-3p mimic 4 h after middle cerebral artery occlusion (MCAo) significantly improved stroke outcomes including infarct volume and sensory motor performance. Improvement was not observed when miR-20a-3p was given immediately or 24 h after MCAo, identifying a unique *delayed* therapeutic window. Overall, this study identifies a novel neuroprotective microRNA and characterizes several key pathways by which it can improve stroke outcomes.

**Supplementary Information:**

The online version contains supplementary material available at 10.1007/s12975-021-00945-x.

## Introduction

Stroke remains the leading cause of disability and the fifth leading cause of mortality in the USA. Ischemic stroke accounts for 80% of all strokes, and there is currently only one FDA-approved drug therapy, tissue plasminogen activator (tPA). tPA must be administered within a short time window of 3–4.5 h to be effective, which limits the proportion of patients eligible to receive this treatment (5.9–7.0%) [1, 2]. Therefore, there is an urgent need for the development of effective and safe therapeutics. Moreover, since stroke risk and stroke severity are modified by the age and biological sex of the patient, it is imperative for preclinical studies of potential stroke therapies to address both variables.

Epigenetic modifiers, such as small non-coding RNAs, have emerged as powerful candidates for several diseases including cancers and immune, infectious, cardiovascular, and neurodegenerative diseases [3–6]. MicroRNAs (miRNAs) are typically 18–25 nucleotides long and bind to complementary sequences in the 3′ UTR of multiple target mRNAs to regulate gene expression [7]. In the case of ischemic stroke, several studies have now shown that miRNA mimics or antagomirs can regulate acute and chronic stroke outcomes [8–12]. Previous work from our lab has shown that the efficacy of miRNA treatment may be restricted to specific age and sex groups. For example, anti-Let7f treatment after middle cerebral artery occlusion (MCAo) improved stroke outcomes in adult female rats but not older females or adult males [8, 13]. Similarly, miR-363-3p, identified by miRNA profiling of serum, modifies stroke outcomes only in females (adult and middle-aged) but not age-matched males [14]. In order to identify a more universally effective microRNA treatment, the present study used a novel approach by focusing on astrocytes.

Astrocytes are a crucial cell type for mediating brain energy homeostasis, providing neurotrophic support, and maintaining the blood–brain barrier [15]. Astrocytes have been shown to develop an “aging” phenotype, characterized by increased glial fibrillary acidic protein (GFAP) expression [16] and increased production of senescence-associated secretory phenotype (SASP) factors, such as interleukins (IL-1α [17], IL-6 [18, 19], IL-8 [20], IL-15 [21]) and matrix metalloproteases (MMP-1 [18], MMP-3, MMP-10 [20]). Astrocytes harvested from the ischemic cortex and striatum in middle-aged females show similar senescence-related changes, including reduced glutamate reuptake, decreased growth factor release, and increased chemokine release compared to adult females, all indicative of decreased neuroprotective capacity [22]. Furthermore, astrocytes from middle-aged females showed reduced trimethylation of histone H3K4, a transcriptional enhancer, as compared to adult females [23], indicating a global reduction in transcription potential, including the transcriptional start site for the miR-17a-92a cluster.

To assess the role of astrocytic miRNA in stroke outcomes, we compared miRNA profiles in astrocytes obtained from the ischemic forebrain of adult and middle-aged males and females. These groups were specifically selected because stroke outcomes are more severe in adult males and middle-aged males and females as compared to adult females, where stroke-induced infarction and sensory motor impairment is low. This approach confirmed the suppression of the miR-17–92 cluster observed in ChIP-seq analysis [23] and further identified miR-20a-3p as a uniquely age- and sex-regulated miRNA, whose expression was dramatically elevated in adult females as compared to all other groups. Bioinformatics identified mitochondrial and inflammation-associated genes as targets of miR-20a-3p, and select examples were confirmed in molecular and functional assays. A viral construct designed to conditionally elevated miR-20a-3p in astrocytes partially improved stroke outcomes. Subsequently, intravenous injection of fluorescently labeled miR-20a-3p after stroke was found to be preferentially taken up by neurons. A second viral construct was designed to conditionally express miR-20a-3p in neurons, resulting in robust neuroprotection. Finally, i.v. treatment of miR-20a-3p, a more translationally viable route of administration, was found to be neuroprotective in both middle-aged males and females. Together, these data indicate that while miR-20a-3p is produced by and profoundly alters the function of astrocytes after ischemia, stroke neuroprotection via this microRNA may depend on other cell types such as neurons receiving and utilizing miR-20a-3p.

## Results

### Expression of MicroRNA from the miR-17–92 Cluster in Astrocytes from Adult and Middle-Aged Males and Females

**Fig. 1:**
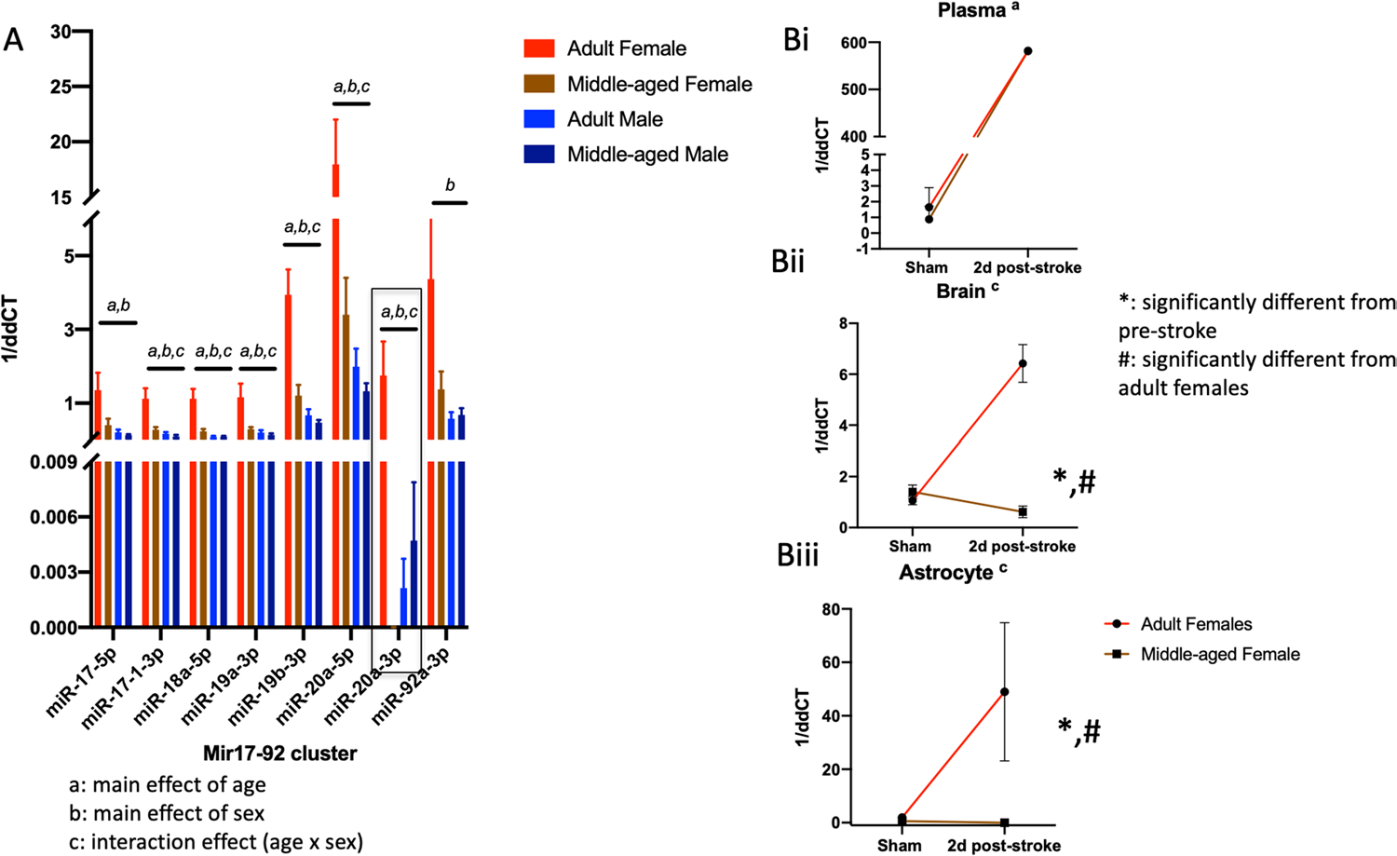
MiRnome analysis of miR expression from the miR-17–92 cluster. A histogram depicting the mean (± SEM) expression of microRNA from the miR-17–92 cluster in adult and middle-aged males and females. Bar above each set indicates main and interaction effects from two-way ANOVAs. *a* Main effect of age. *b* Main effect of sex. *c* Interaction effect (age × sex). B MiR-20a-3p expression in serum, whole brain, and astrocyte. Pre-stroke and 2-day post-stroke serum, brain, and astrocyte samples were evaluated for miR-20a expression. Key: asterisk, significantly different from pre stroke; number sign, significantly different from adult females at that time point. *p* < 0.05. *n* = 6 in most groups, *n* = 3 in pre-stroke astrocytes and post stroke brain samples. Red line, adult females; black line, middle-aged females

Astrocytes were extracted at 48 h post stroke from adult and middle-aged females and males using positive selection for GLAST. Based on our previous data showing increased trimethylation of the miR-17–92 cluster in adult females, we analyzed sex and age differences in this cluster by qRT-PCR (normalized to U6). Members of this miRNA cluster showed a complex regulation due to age and sex. As shown in Fig. 1a, miR-17-5p, miR-18a-5p, miR-19a, miR-19b, miR-20a-5p, and miR-20a-3p displayed a significant age × sex effects, such that each of these miRNAs was elevated in the adult female as compared to the adult male and middle-aged male or female. In the case of miR-17-3p, there were a main effect of age and a main effect of sex, while in the case of miR-92a-3p, there was a main effect of age, where adult animals had a higher expression of these miRNAs as compared to middle-aged animals. The most remarkable age × sex regulation was seen in miR-20a-3p expression, which demonstrated *a* > 240,000-fold elevation in adult female as compared to middle-aged females. A separate analysis of the entire miRnome panel controlling for false discovery rate (FDR) showed that miR-20a-3p was the only astrocytic miRNA that was significantly elevated by age and sex.

### miR-20a-3p Expression in the Circulation, Brain, and Astrocytes in Adult and Middle-Aged Females

To determine if miR-20a-3p suppression is a tissue-specific response to stroke, miR-20a-3p expression was analyzed using qRT-PCR in serum, whole-brain homogenate, and astrocytes from adult and middle-aged females in sham animals and at 2 days post stroke. While basal expression of miR-20a-3p is similar at both ages, miR-20a-3p is altered in an age- and tissue-specific manner (Fig. 1b) during the early acute phase (2 days) of stroke. In circulation (serum), miR-20a-3p is significantly elevated at 2 days post stroke in both adult and middle-aged females (Fig. 1b(i)). In whole-brain homogenate (Fig. 1b(ii)) and in astrocytes (Fig. 1b(iii)), miR-20a-3p expression is preferentially elevated in adult females (red line) and profoundly suppressed in middle-aged females (black line) at 2 days post stroke.

### Effect of MiR-20a-3p Treatment on Astrocyte Mitochondrial Function In Vitro

**Fig. 2:**
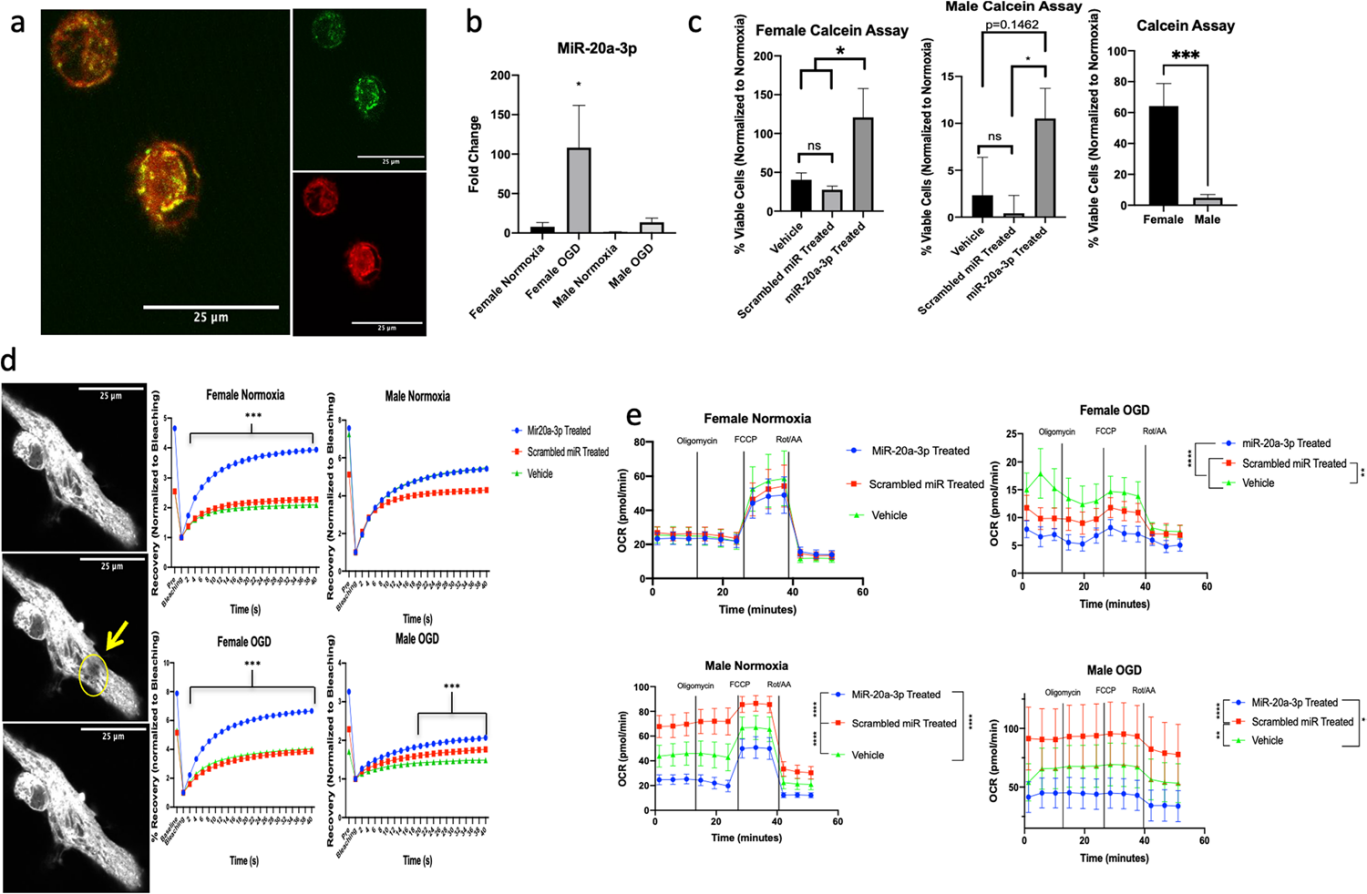
Effect of miR20a-3p mimic on mitochondrial dynamics. **a** Primary human astrocytes stained with MitoTracker Deep Red and incubated with FAM-labeled miR-20a-3p for 6 h. **b** qRT-PCR expression of miR-20a-30 in primary human astrocytes. **c** Calcein assay on female and male human astrocytes. Histogram depicting the mean (± SEM) percent of viable cells relative to normoxic conditions. **d** FRAP analysis of female and male human astrocytes. The recovery was normalized to the level of bleaching in the cells (bleaching value was set at 1). **e** Seahorse XFe96 Mito Stress Test on human astrocytes. *p ≤ 0.05, **p ≤ 0.01, ***p ≤ 0.001, ****p ≤ 0.0001

Bioinformatics analyses (using databases TargetScan and MiRWalk 2.0) indicate that miR-20a-3p regulates a large number of genes that are responsible for mediating mitochondrial function (Supplementary Fig. 1). In order to investigate specific mechanisms by which miR-20a-3p alters mitochondrial function, male and female human astrocytes were cultured and subjected to 6 h of oxygen glucose deprivation (OGD) (1% O_2_, 0 mM glucose) or normoxia (21% O_2_, 25 mM glucose) and treated with miR-20a-3p mimic, scrambled miR, or vehicle. Astrocyte cultures were confirmed by immunohistochemistry for GFAP (green) and co-labeled with nuclear dye DAPI (blue) (Supplementary Fig. 2a). Cultures that were incubated with 50 nM FAM-labeled miR-20a-3p mimic and MitoTracker Deep Red to stain living mitochondria for 6 h showed that the microRNA is readily taken up by astrocytes (Fig. 2a). In addition, qRT-PCR analysis indicates that female human astrocytes profoundly upregulate miR-20a-3p in response to ischemia, whereas the male astrocytes do not (Fig. 2b), recapitulating our in vivo observations.

To determine the effect of miR-20a-3p on astrocyte survival after OGD, cells were incubated with Calcein-AM dye (Life Technologies, CA) after OGD treatment. MiR-20a-3p treatment significantly increased cell viability in both male and female cells compared to scrambled miR treatment, but with different degrees of efficacy. In females, cell viability in the miR-20a-3p treatment group was virtually similar to that in normoxic controls, while cell viability in males that received miR-20a-3p was greater than that in cultures that received the scrambled oligo but was far lower than that in normoxic controls. Moreover, the female astrocytes demonstrated a greater degree of viability as compared to the male astrocytes after exposure to OGD (*p* = 0.002, Fig. 2c).

Two functional assays were used to explore the effect of miR-20a-3p treatment on astrocytic mitochondrial function. The first technique used was fluorescence recovery after photobleaching (FRAP), in which human astrocyte cultures were labeled with MitoTracker Deep Red and a small area of the cell was photobleached with a high intensity 405-nm laser and the subsequent fluorescent recovery was quantified using ImageJ. MiR-20a-3p treatment significantly increased recovery in both OGD and normoxic female groups immediately after bleaching (< 4 s), while the miR-20a-3p—treated male cells only exhibited significantly increased recovery in the OGD group after 18 s (Fig. 2d). Another functional assay used to assess mitochondrial dynamics is the Mito Stress Test using the Seahorse 96XFe Analyzer. This assay involves the serial injection of various drugs (oligomycin, FCCP, and rotenone + antimycin A) that target components of the electron transport chain. In normoxic conditions, female astrocytes show no difference between the miR-20a-3p—treated group and either of the control groups; however, the miR-20a-3p—treated male astrocytes show reduced oxygen consumption rate relative to both vehicle and scrambled oligo–treated cells (Fig. 2e). Moreover, miR-20a-3p treatment significantly reduces oxygen consumption rates in the male and female astrocytes in OGD conditions, though the male cells exhibit significantly more variability than the female cells (Fig. 2e). Increased oxygen consumption could be indicative of cell senescence induced by oxidative damage [24], and these data suggest that miR-20a-3p may reduce this senescent feature in an ischemic environment.

### Astrocyte-Specific Enhancement of MiR-20a-3p Improves Select Stroke Outcomes in Middle-Aged Females

**Fig. 3:**
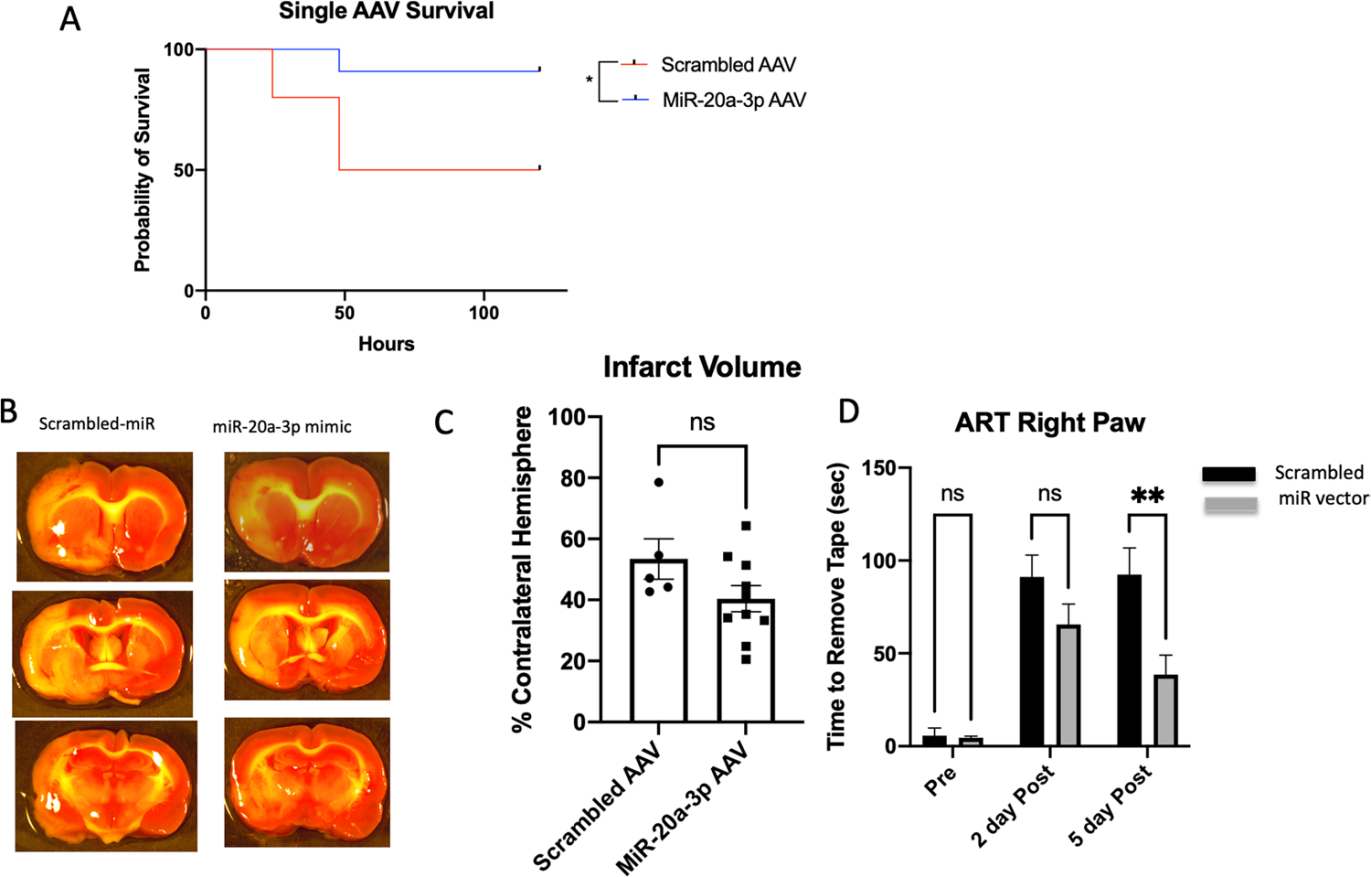
Effect of rAAV-TetOn-GFAP-miR-20a-3p on stroke outcome in middle-aged females. A Kaplan–Meier survival plot shows significantly greater mortality in animals that received the control vector as compared to the miR-20a-3p–containing vector. B Representative images of TTC-stained sections obtained 5 days post MCAo. C Histogram depicting mean (± SEM) infarct volume. D Histogram of mean (± SEM) latency to remove the adhesive tape pre MCAo, 2 days post MCAo, and 5 days post MCAo. *N* = 12 (miR-20a-3p) and *n* = 11 (scrambled oligo) pre MCAo. *p ≤ 0.05, **p ≤ 0.01

To assess the neuroprotective potential of astrocyte-derived miR-20a-3p in the context of stroke, a viral construct (rAAV5-TetOn-GFAP-miR-20a-3p-mCherry, SignaGen) under the control of a TetOn system with a GFAP promoter and conjugated with a mCherry reporter (Supplementary Figure 3a) and a control vector (Supplementary Figure 3b) was synthesized and injected into the left hemisphere of middle-aged female rat brains. Figure 3c demonstrates that the recombinant adeno-associated virus (rAAV) is activated by doxycycline and primarily co-localizes with GFAP. Two doses (2.5 × 10^11^ VP/ml; 2.5 × 10^10^ VP/ml) of the rAAV were used in pilot studies to determine the most effective dose to assess the effect of astrocyte-specific miR-20a-3p expression after stroke. Striatal injections of the high dose (2.5 × 10^11^ VP/ml) did not result in any adverse effects on body weight or spleen weight and were analyzed extensively for stroke outcomes (Fig. 3a–e). The Kaplan–Meier survival analysis (Fig. 3a) showed that the striatal injection of the rAAV containing miR-20a-3p reduced stroke-induced mortality (*p* = 0.0372). Infarct volume (Fig. 3b, c) assessed at 5 days after MCAo in animals that received the control vector was greater (53.42%) than that in animals that received the miR-20a-3p—containing vector (40.39%), although this difference did not meet the criteria for statistical significance (*p* = 0.1117). Sensory motor performance assessed by the adhesive removal test showed that both groups had increased latency at 2 days post stroke on the contralesional limb, while at 5 days post stroke, the miR-20a-3p group showed improved recovery and significantly reduced latency as compared to the control vector group (Fig. 3d). The vibrissae-evoked forelimb placement task did not demonstrate any improvement in the percentage of correct responses post MCAo when the same-side vibrissae were stimulated nor when the cross-midline vibrissae were stimulated (Supp. Fig. 4A).

### FAM-Labeled miR-20a-3p Localizes to Neurons Rather Than Astrocytes After MCAo

**Fig. 4:**
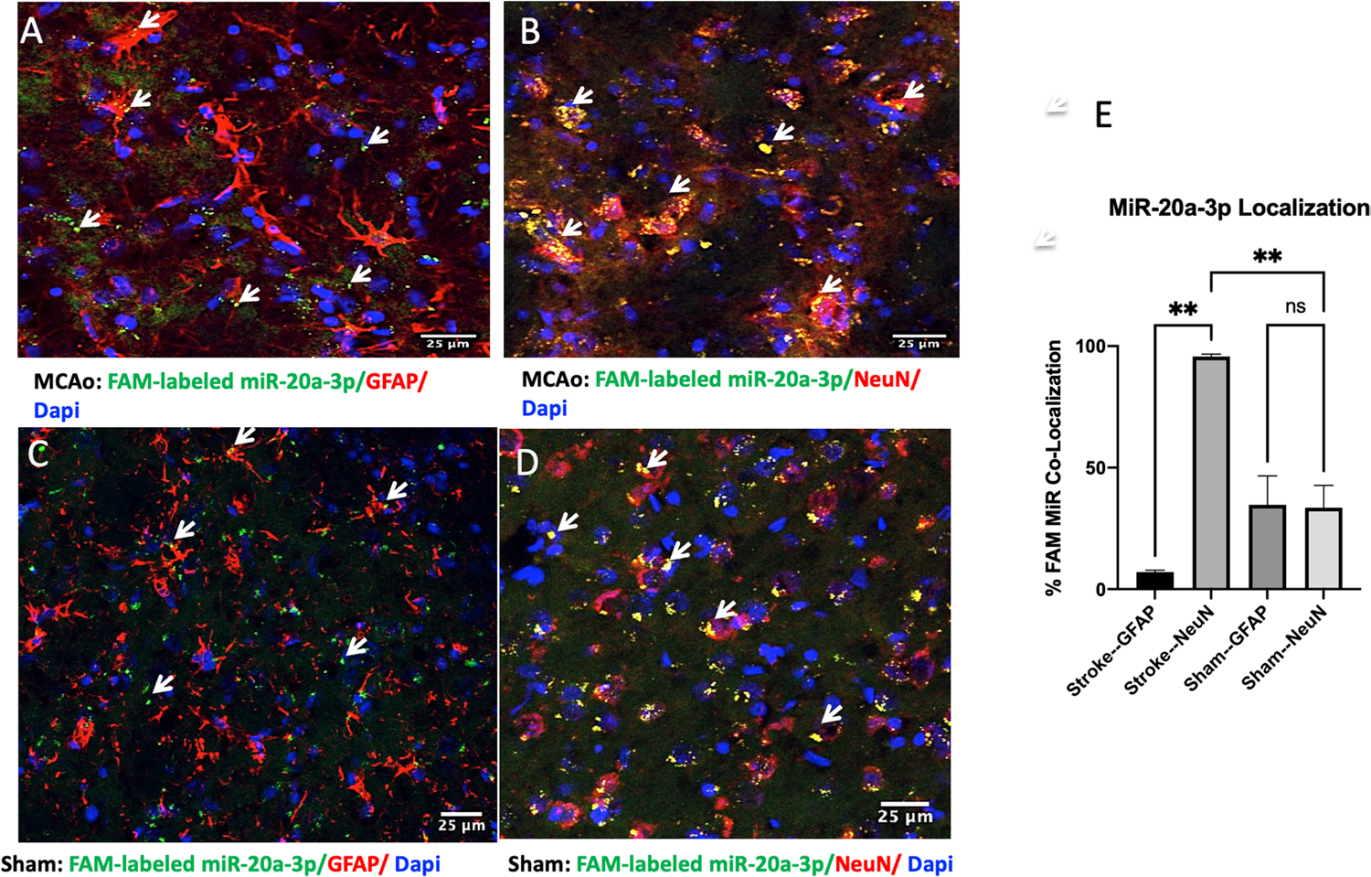
Cellular localization of i.v. miR-20a-3p. Photomicrographs of coronal sections from animals injected with FAM-labeled miR-20a-3p after MCAo or sham surgery probed for glial and neuronal markers and counterstained for DAPI (blue). A MCAo: GFAP (red). B MCAo: NeuN (red). C Sham: GFAP (red). D Sham: NeuN (red). Arrows indicate FAM-labeled miR-20a-3p (green). E Quantification of FAM-miR-20a-3p co-localization. **p ≤ 0.01

We next examined the efficacy of intravenously injected miR-20a-3p. First, a FAM-labeled miR-20a-3p mimic was injected via tail vein 4 h after MCAo or sham surgery. The FAM-labeled mimic was chosen in order to visualize the localization of the miR-20a-3p mimic after intravenous treatment. FAM-labeled miR-20a-3p (green) preferentially co-localized with NeuN+ cells rather than GFAP+ cells in the animals that received MCAo (Fig. 4a, b). The proportion of FAM-labeled miR-20a-3p expressed in cells (co-labeled with dapi) that co-localized with NeuN+ cells compared to GFAP+ cells confirmed that neurons preferentially uptake the FAM-labeled miR (Fig. 4e). Interestingly, in animals that received the sham surgery, this preferential uptake by neurons is not observed (Fig. 4c–e). These data indicate that neurons may uptake this miRNA more readily under ischemic conditions.

### Effect of MiR-20a-3p on Neuronal Mitochondrial Function In Vitro

**Fig. 5:**
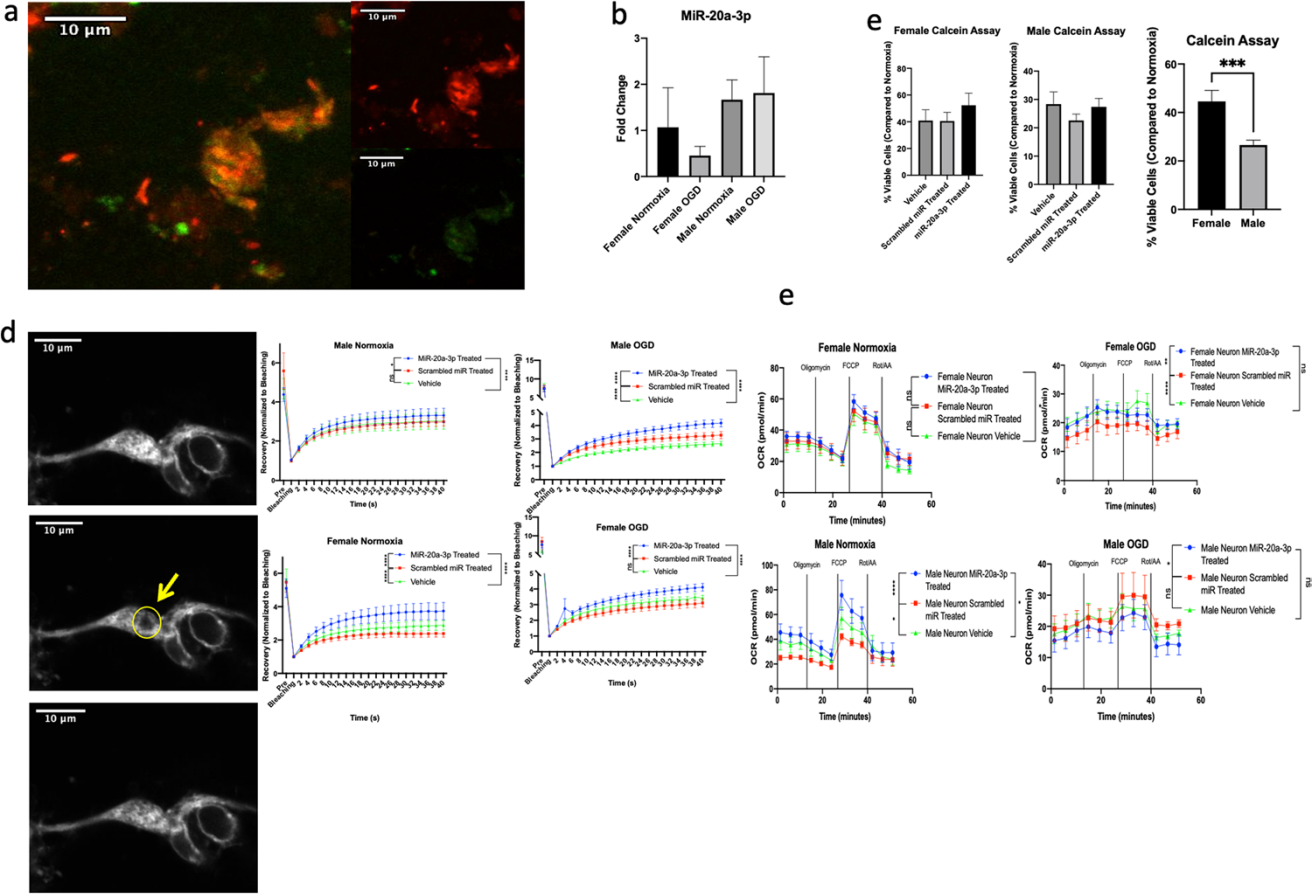
Effect of miR20a-3p mimic on neuronal mitochondrial dynamics. **a** Primary human neurons stained with MitoTracker Deep Red and incubated with FAM-labeled miR-20a-3p for 30 min. **b** qRT-PCR expression of miR-20a-30 in primary human neurons. **c** Calcein assay on female and male human neurons. Histogram depicting the mean (± SEM) percent of viable cells relative to normoxic conditions. **d** FRAP analysis of female and male human neurons. The recovery was normalized to the level of bleaching in the cells (bleaching value was set at 1). **e** Seahorse XFe96 Mito Stress Test on human neurons. *p ≤ 0.05, **p ≤ 0.01, ***p ≤ 0.001, ****p ≤ 0.0001

In light of the cellular localization of i.v. FAM-miR-20a-3p injections, we decided to investigate the effects of miR-20a-3p on neuronal mitochondrial functions. Primary human neurons were cultured in identical conditions to the previously described astrocytes. Neuronal identify was confirmed via immunohistochemistry for NeuN (red) and co-labeled with the nuclear stain DAPI (Supplementary Fig. 2b). OGD was reduced to 30 min to account for greater cell death of the neurons relative to astrocytes in ischemic conditions. Confocal images indicate that the neurons uptake the FAM-labeled miR-20a-3p mimic after 30 min of OGD (Fig. 5a). qPCR analysis was performed to assess the expression of miR-20a-3p in normoxic and OGD conditions, and no differences in the expression of this microRNA were observed in either sex (Fig. 5b).

A cell viability assay, FRAP analysis, and the Seahorse Mito Stress Test assay were performed to assess the effect of miR-20a-3p on neuronal mitochondrial function. The cell viability assay did not show any improvement of neuronal survival after 30 min of OGD, though the female neurons did demonstrate a greater degree of viability as compared to the male neurons (*p* = 0.0007, Fig. 5c); however, key functional differences were elucidated in the FRAP and Seahorse assays. In all cases, neurons treated with miR-20a-3p significantly improved fluorescent recovery after photobleaching (Fig. 5d), similar to what was observed in the astrocytes. The Seahorse assay, however, demonstrated differences between neurons and astrocytes regarding the effect of miR-20a-3p on oxygen consumption. In normoxic conditions, male neurons treated with miR-20a-3p showed significantly elevated levels of oxygen consumption rate relative to control groups (Fig. 5e). The female neurons treated with miR-20a-3p exhibited greater oxygen consumption relative to vehicle, but there was not a significant difference between miR-20a-3p-treated and scrambled miR–treated groups. OGD reduced the rate of oxygen consumption in both sexes, though the difference between the miR-20a-3p–treated groups and the vehicle groups is not significant (Fig. 5e).

### Neuron-Specific Enhancement of MiR-20a-3p is Neuroprotective in Middle-Aged Female Rats After Stroke

**Fig. 6:**
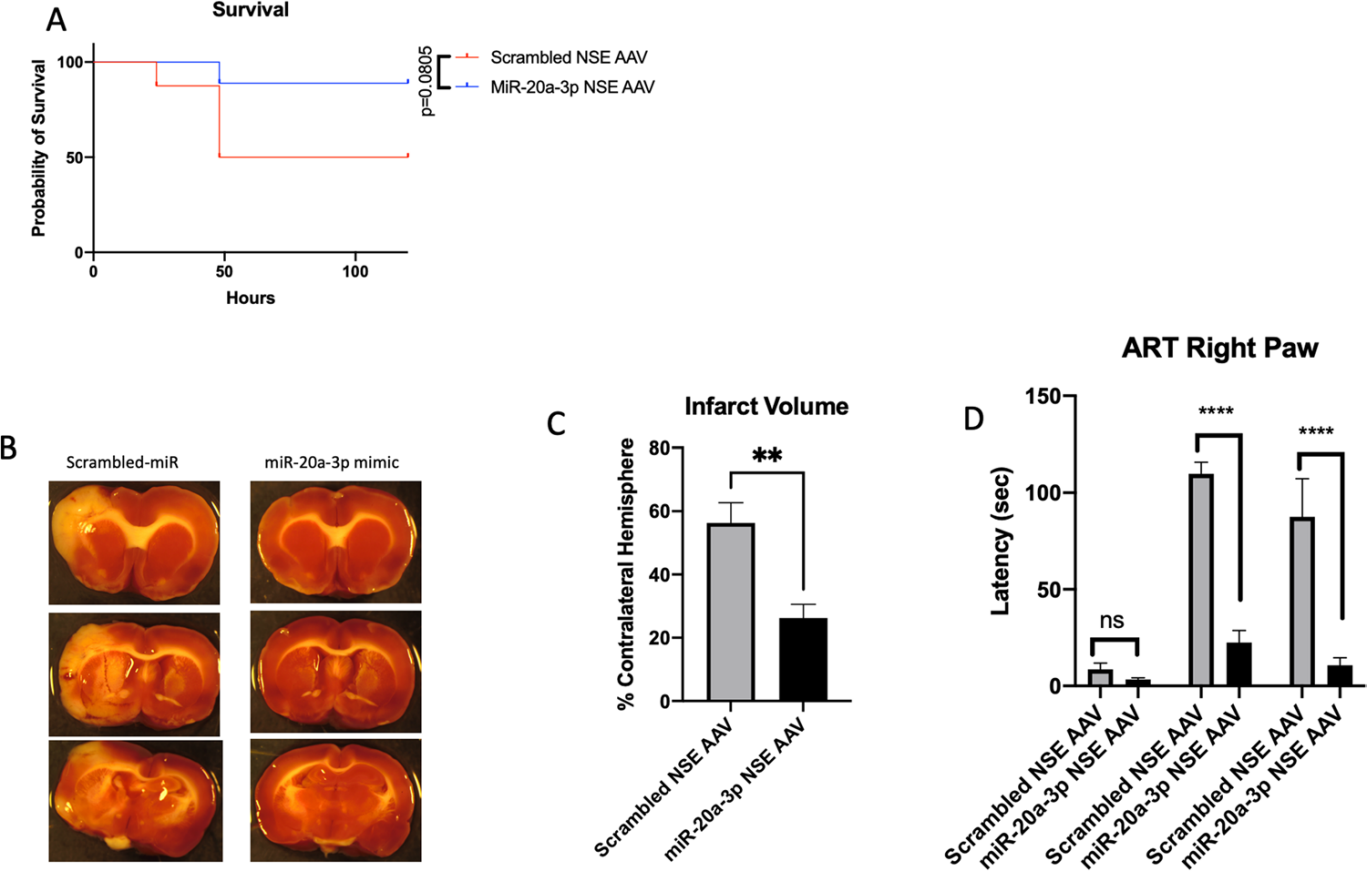
Effect of rAAV-TetOn-NSE-miR-20a-3p on stroke outcome in middle-aged females. **a** Kaplan–Meier survival plot shows a trend toward greater mortality in animals that received the control vector as compared to the miR-20a-3p—containing vector. **b** Representative images of TTC-stained sections obtained 5 days post MCAo. **c** Histogram depicting mean (± SEM) infarct volume. **d** Histogram of mean (± SEM) latency to remove the adhesive tape pre MCAo, 2 days post MCAo, and 5 days post MCAo. *N* = 10 (miR-20a-3p) and *n* = 9 (scrambled oligo) pre MCAo. *p ≤ 0.01, ****p ≤ 0.0001

A second viral construct (rAAV5-TetOn-NSE-miR-20a-3p mCherry, SignaGen) was created to assess the effect of conditional neuronal miR-20a-3p expression. rAAV striatal injections and endothelin-1 (ET-1) surgeries were performed as indicated previously, and all procedures were identical to the previous rAAV experiment. However, unlike the GFAP vector, the neuron-specific enolase (NSE) vector showed significant reduction of infarct volume in animals that received the rAAV vector (Fig. 6b, c, p = 0.0087) and more significant improvement in the sensory motor tasks at both 2 days and 5 days post stroke (Fig. 6d, *p* < 0.0001). While post-stroke mortality in the rAAV-NSE-miR-20a-3p was almost half that of the group receiving the control vector, the Kaplan–Meier survival analysis only indicated a statistical trend in survival (Fig. 6a, *p* = 0.0805).

### MiR-20a-3p Treatment Significantly Reduced Infarct Volume in Middle-Aged Female and Male Rats

**Fig. 7:**
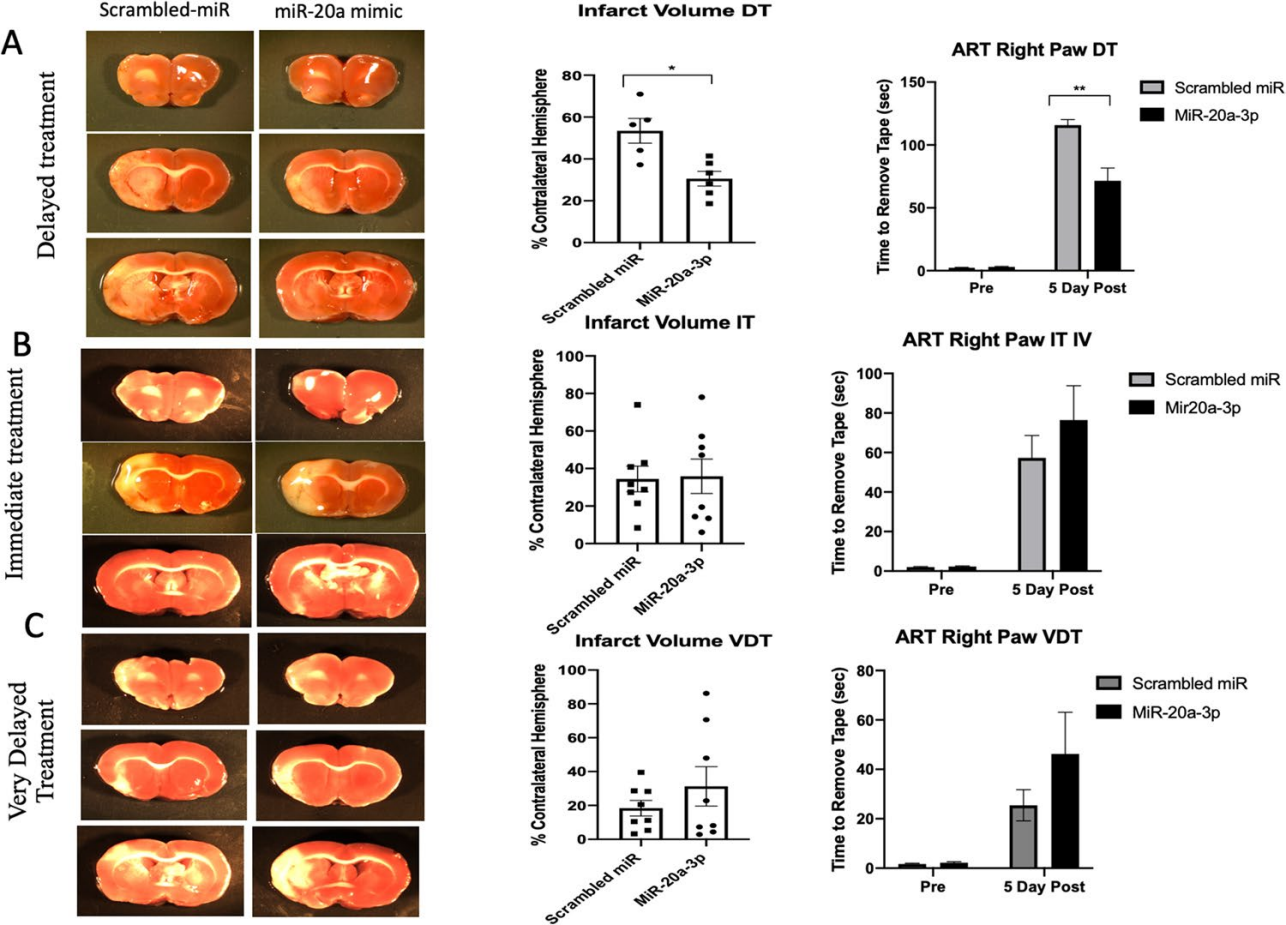
Effect of intravenous miR-20a-3p mimics on stroke outcomes in middle-aged females at IT, DT, and VDT: **A**(i)–**C**(i) TTC-stained coronal sections from middle-aged females treated with scrambled oligo or miR-20a-3p. **a**(ii)–**c**(ii) Histogram depicts average infarct volume (± SEM) normalized to the volume of the non-ischemic hemisphere. **a**(iii)–**c**(iii) Sensory motor performance on the adhesive removal test was evaluated before and after stroke. Histograms depict mean (± SEM) latency in seconds to remove the tape. DT, *N* = 6 (control or treatment); IT, *N* = 7 (control) and *N* = 8 (treatment); VDT, *N* = 8 (control) and *N* = 8 (treatment). *p ≤ 0.05, **p ≤ 0.01

Although the rAAV experiments provide key insight into the cellular mechanism of neuroprotection of miR-20a-3p, i.v. treatment is much less invasive and therefore more translationally viable, so we next assessed the effect of i.v. miR-20a-3p on acute stroke outcomes. Animals were subjected to MCAo and injected via the tail vein with miR-20a-3p mimic either concurrent (immediate treatment (IT)) with the onset of ischemia, 4 h after ischemic onset (delayed treatment (DT)), or 24 h after the onset of ischemia (very delayed treatment (VDT)). As shown in Fig. 7a, triphenyl tetrazolium chloride (TTC)-stained images indicate that the cortico-striatal infarct is significantly reduced in middle-aged females in animals that received miR-20-3p 4 h after MCAo (DT) as compared to the age-matched counterparts that received i.v. injections of the scrambled oligos. Delayed miR-20a-3p treatment decreased infarct volume by 60% and improved performance on the adhesive removal test when measured 5 days post stroke (Fig. 7a). On the other hand, i.v. injections of miR-20a-3p–delivered concurrent with the onset of ischemia (IT) or 24 h after MCAo (VDT) did not show any decrease in infarct size or reduction in sensory motor deficits (Fig. 7b, c, Supp. Fig. 4c, d).

Middle-aged males (a group that also sustains severe stroke outcomes) showed significantly reduced infarct following delayed miR-20a-3p mimic treatment (Supplementary Fig. 3A, B; *p* < 0.05) and significantly improved performance on the adhesive removal test, which is impaired after MCAo (Supplementary Fig. 3C). Collectively, these data indicate that, similar to middle-aged female rats, i.v. miR-20a-3p mimic to middle-aged male rats improved stroke outcomes.

### Effect of DT MiR-20a-3p Treatment on Gene Targets Post MCAo

**Fig. 8:**
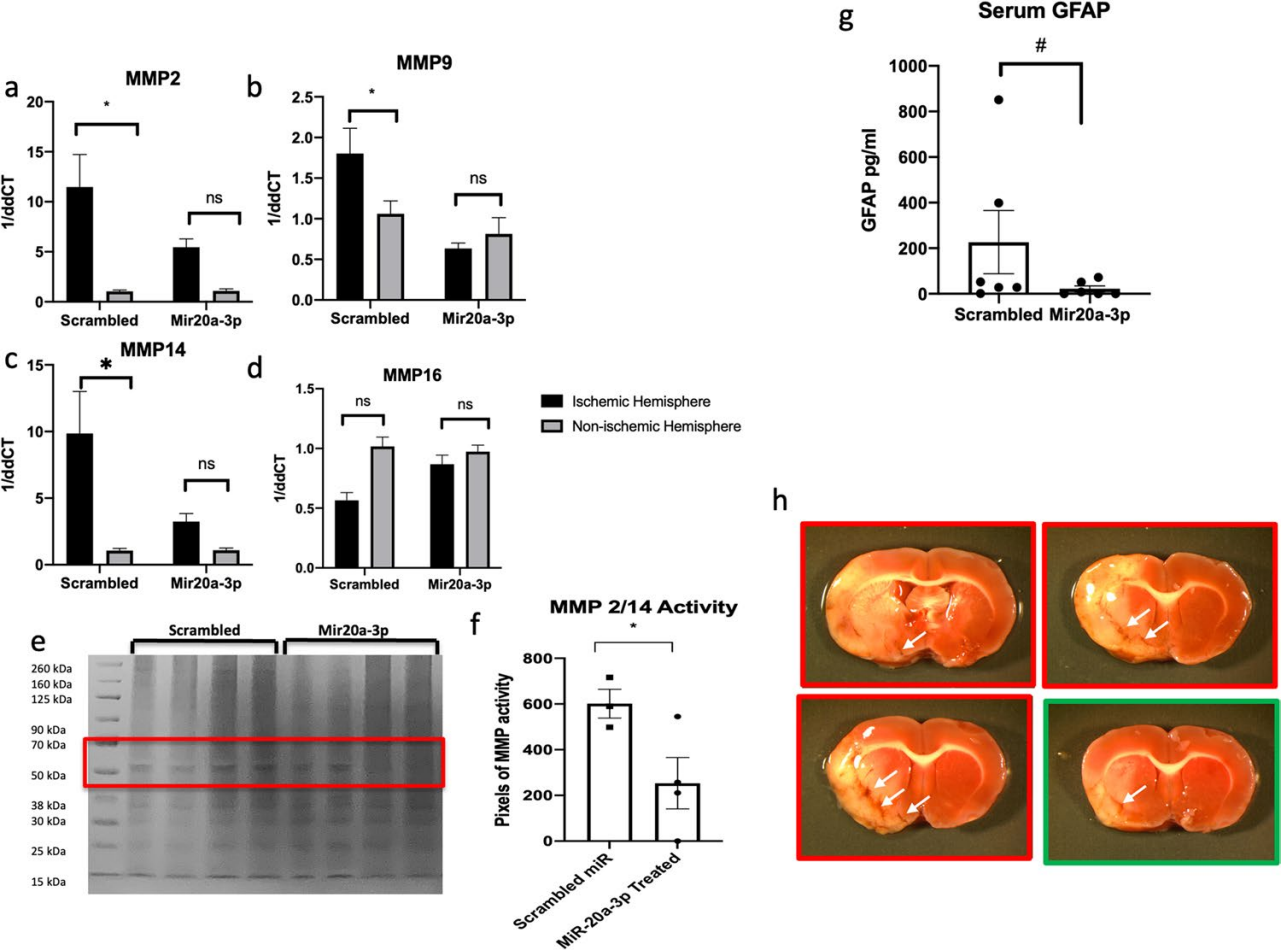
Effect of intravenous miR-20a-3p on markers blood–brain barrier permeability. **a**–**d** qPCR analysis of MMPs from the ischemic and non-ischemic hemispheres of scrambled and miR-20a-3p–treated animals. **e** Representative image of gelatin Zymogram depicting MMP activity from protein lysates from the ischemic hemisphere. **f** Histogram showing the mean (±) of MMP activity from gelatin zymography. **g** ELISA analysis of GFAP expression in serum. **h** Representative TTC images of brain slices with hemorrhagic foci from females treated with miR-20a-3p mimic and scrambled oligo. Foci are indicated by white arrows. *N* = 6 (miR-20a-3p) and *n* = 6 (scrambled oligo). ^#^*p* < 0.1, *p ≤ 0.05

To assess whether i.v. treatment of miR-20a-3p affected astrocyte gene expression, astrocytes were isolated from the ischemic hemisphere for qRT-PCR analysis. In view of the improved stroke outcomes in the DT group, the analysis was only performed in this group. Several matrix metalloproteinases (MMPs) and mitochondrial gene targets of miR-20a-3p demonstrated altered regulation in the ischemic hemisphere of the animals that received DT as compared to the animal that received scrambled treatment. Gene expression of MMP-2, MMP-9, and MMP-14 was elevated in the ischemic hemisphere of animals that received scrambled oligo treatment, while this elevation was mitigated in the ischemic hemisphere of animals receiving miR-20a-3p (Fig. 8a–c, *p < 0.05). MMP activity was assessed by gelatin zymography. Protein was isolated from the ischemic hemisphere of animals subjected to MCAo that received either miR-20a-3p mimic or scrambled oligo (DT). Lytic activity was noted in the region between 50 and 70 kDa (Fig. 8e), which contains MMP-2 and MMP-14. This region was quantified for densitometry by ImageJ and revealed a significant reduction in MMP activity in the miR-20a-3p—treated animals (Fig. 8f). MMPs have been implicated in the blood–brain barrier (BBB) and cerebral microvasculature damage as well as hemorrhagic bleeds [25]. BBB damage was assessed by ELISA for serum GFAP, which is a surrogate measure of BBB leakiness. As shown in Fig. 8g, there was a trend (*p* = 0.12) toward decreased GFAP levels in the miR-20a-3p—treated groups. Hemorrhagic foci were inspected in TTC-stained sections (shown in Fig. 8h) by a code-blinded observer and were found to be present in all (100%) scrambled oligo-treated middle-aged female rats and in one-third of the miR-20a-3p—treated rats (chi-square; *p* < 0.0143), generally in the vicinity of striatal vessels. Collectively, these data suggest that miR-20a-3p treatment 4 h after MCAo may protect the brain against MMP-mediated BBB damage and subsequent hemorrhagic transformation.

**Fig. 9:**
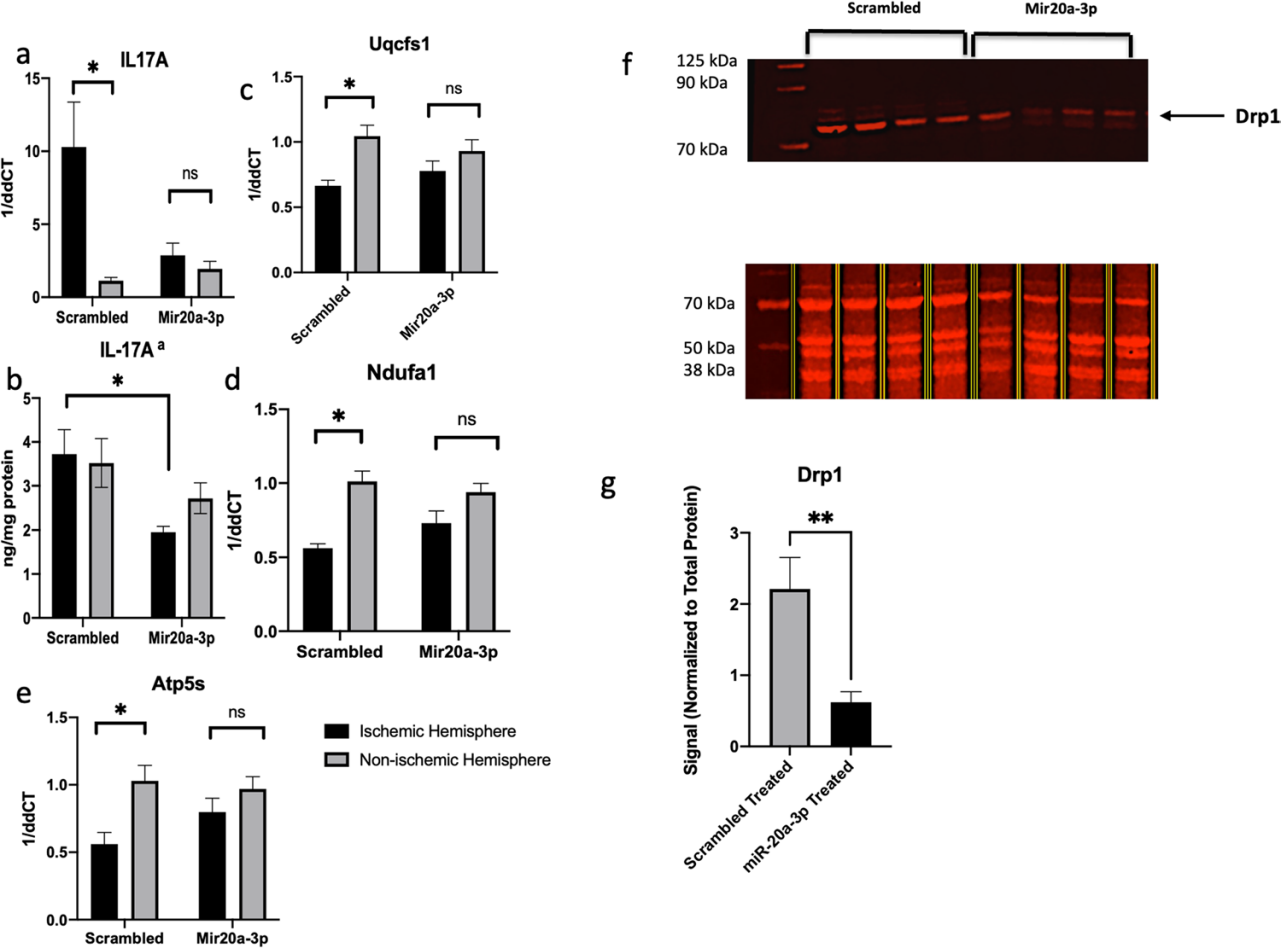
Effect of intravenous miR-20a-3p on predicted mitochondrial targets. **a** qPCR and **b** ELISA analysis of IL-17A expression from the ischemic and non-ischemic hemispheres of scrambled and miR-20a-3p–treated animals. **c**–**e** qPCR analysis of predicted mitochondrial genes from the ischemic and non-ischemic hemispheres of scrambled and miR-20a-3p–treated animals. **f** Protein from the ischemic hemisphere of scrambled and miR-20a-3p–treated animals probed for Drp1 by western blot and total protein stain. **g** Histogram of the mean (± SEM) of Drp1 normalized to total protein. *N* = 6 (miR-20a-3p) and *n* = 6 (scrambled oligo). *p ≤ 0.05, **p ≤ 0.01

IL-17A is a pro-inflammatory cytokine, which is a predicted target of miR-20a-3p and a therapeutic target for anti-inflammatory drugs to improve recovery post stroke [26]. Similar to the MMPs, IL-17A was elevated in the ischemic hemisphere of animals that received scrambled oligo treatment, while this elevation was mitigated in the ischemic hemisphere of animals receiving miR-20a-3p (Fig. 9a). This was further confirmed by ELISA assay, showing that IL-17A expression was significantly reduced in the ischemic hemisphere of the miR-20a-3p–treated middle-aged females as compared to the scrambled controls (Fig. 9b, p ≤ 0.05). Circulating (serum) levels of IL-17A were not different in the two groups (data not shown), suggesting that miR-20a-3p either has a brain-specific effect for this cytokine or regulates the blood–brain barrier to prevent extravasation of IL-17A or IL-17A–producing cells.

Mitochondrial gene targets including Uqcfs1, Ndufa1, and Atp5s were reduced in the ischemic hemisphere of the scrambled oligo–treated animals, while there were no differences in the expression of these genes in the two hemispheres of the miR-20a-3p–treated animals (Fig. 9c–e), indicating that miR-20a-3p treatment preserved the expression of mitochondrial genes. Quantitative western blot analysis was performed on protein isolated from the ischemic hemisphere of animals subjected to MCAo that received DT of either miR-20a-3p mimic or scrambled oligo. The blot was probed with a Drp1 (mitochondrial fission protein) antibody (Fig. 9f) and normalized to the LiCOR Total Protein Stain. MiR-20a-3p–treated animals demonstrated significantly less expression of Drp1 as compared to scrambled oligo–treated animals (Fig. 9g).

## Discussion

The present study used well-known age and sex differences in stroke outcomes to identify microRNA with neuroprotective potential. We observed that the miR-17–92 cluster, which has been shown to improve neural plasticity after stroke in male rats/mice [27], was significantly reduced in adult males and middle-aged males and females, groups that typically exhibit poor stroke outcomes, compared to adult females, a group that typically exhibits minimal stroke impairment. Moreover, one member of this miRNA cluster, miR-20a-3p, was dramatically elevated in young females, primarily in astrocytes. In view of the fact that young females sustain smaller infarcts and recover better than other demographics, subsequent studies focused exclusively on this microRNA. These studies used three methods of delivery: astrocyte-specific miR-20a-3p, neuron-specific miR-20a-3p, and i.v. injections of miR-20a-3p. MiR-20a-3p altered mitochondrial function in astrocyte and neuronal cultures, and conditional elevation of this miRNA in astrocytes in middle-aged females partially improved stroke recovery, while conditional expression of miR-20a-3p resulted in robust neuroprotection. Moreover, i.v. injections of miR-20a-3p, which is a more tractable therapeutic approach, also improved infarct volume and sensory motor behavior.

Astrocytes play an important role as neuronal support cells in a variety of ways, a critical one being the maintenance of the BBB [28], which protects the brain’s exposure to pathogens and immune cells. Astrocytic end-feet located on the basal lamina surrounding brain microvessels are a critical physical and biochemical barrier. Destruction of this endothelial basal lamina is an early cause of hemorrhage after focal cerebral ischemia [29], resulting from increased activation of MMPs. MMPs, a group of proteolytic zinc-dependent enzymes, are elevated during stroke in humans [30] and in experimental models of focal ischemia [31] and directly contribute to edema and hemorrhage [32]. Coupled with the loss of endothelial tight junction proteins, elevated levels of MMPs further deteriorate the BBB, augmenting neuroinflammation and cell death. Thus, reduced transcriptional and functional MMP levels may underlie the decreased hemorrhagic foci and robust neuroprotective effects seen in groups treated with miR-20a-3p.

In view of the diverse predicted miR-20a-3p targets, including mitochondrial genes, there may be multiple potential pathways by which miR-20a-3p promotes neuroprotection post stroke. Mitochondrial dysfunction after ischemic stroke is responsible for much of the damage associated with ischemic injury, resulting in massive cell death [33]; thus, drugs that preserve healthy mitochondrial function are critically pursued as therapeutic options. After stroke, the homeostatic balance between mitochondrial fission proteins and mitochondrial fusion proteins is disrupted, resulting in excessive mitochondrial fission, a feature commonly implicated in a number of CNS diseases including Alzheimer’s disease [34]. Furthermore, cells subject to ischemia must undergo a transition from utilizing oxygen to facilitate energy production to producing energy via other means such as glycolysis [35]. Data from this study indicated that astrocytes treated with miR-20a-3p showed decreased oxygen consumption relative to other treatment groups, though this reduction is not similarly observed in neurons treated with miR-20a-3p. This may indicate that miR-20a-3p’s mechanism of neuroprotection lies in altering metabolic coupling between astrocytes and neurons. This effect in astrocytes may indicate that miR-20a-3p may facilitate effective transition to glycolysis in astrocytes, which may help to facilitate lactate transfer from astrocytes to neurons in ischemic conditions [36]. Alternatively, the reduction in oxygen consumption of the miR-20a-3p—treated astrocytes may simply free up oxygen for other cells such as neurons to consume during ischemic demand. This data supports studies that demonstrate that inhibition of eukaryotic initiation factor 5A hypusination via GC7 treatment, a compound known to reduce basal oxygen consumption, has been shown to be protective after stroke [37] and in an ischemia reperfusion–induced renal injury model [38]. Cellular senescence triggered by oxidative damage has been shown to increase oxygen consumption rate relative to non-senescent cells [24]. Reducing a senescent phenotype in astrocytes may serve to promote neuronal function and survival. Moreover, protein lysates from the brains of rats that were treated with miR-20a-3p after MCAo showed decreased expression of canonical SASP factors, including IL-17A and several MMPs. These data suggest that miR-20a-3p may potentially reduce senescence in these astrocytes, though more studies are needed to confirm this.

In view of their central role in neuronal protection, astrocytic products, including neurosteroids, growth factors, and epigenetic modifiers, are vigorously pursued as stroke therapies. For example, in comparison to any other brain cell, astrocytes produce the most steroids, including progesterone, testosterone, and estradiol, and express enzymes involved in hormone synthesis, including aromatase (Zwain and Yen 1999). An increase in astrocyte-derived estradiol was shown to be neuroprotective and anti-inflammatory following global ischemia, an effect that was blocked by aromatase antisense oligonucleotides (Zhang et al. 2014). Astrocytes also produce multiple growth factors such as VEGF, IGF-1, and BDNF, and decreased expression of these growth factors is a feature of aging astrocytes and a possible explanatory factor for the typically severe stroke outcomes seen in older animals. Our previous work shows that VEGF and IGF-1 are both decreased in the aging astrocyte [22, 23]. Additionally, a rAAV-mediated increase in astrocytic IGF-1 reduced infarct volume and stroke-induced sensorimotor deficits in middle-aged female rats [39]. In the case of miRNAs, astrocyte-enriched species such as miR181 and miR29 that target the Bcl-2 family have also been implicated in reducing ischemic cell death [40, 41]. This study is the first to show that conditional elevation of astrocyte-specific miR-20a-3p improves survival and stroke-induced sensory motor performance, though it had no effect on infarct volume. Moreover, this study also shows that neuron-specific miR-20a-3p was sufficient to significantly improve infarct volume and sensory motor function. The juxtaposition of the fact that astrocytes are the cells that upregulate miR-20a-3p after stroke yet neurons more efficiently utilize the miRNA to improve stroke outcome is interesting. It is possible that this miRNA is specifically transferred from astrocytes to neurons or other neural cell types to confer neuroprotection. Support for this hypothesis comes from a study where extracellular vesicles enriched for the miR-17–92 cluster increase functional recovery after stroke in young male rats [27]. More studies to examine how miR-20a-3p affects neuron-glia interactions are critical to thoroughly understand this interaction.

A unique aspect of this study is the finding that miR-20a-3p treatment was more effective when administered 4 h (delayed) after stroke as opposed to immediately after stroke. Studies of stroke neuroprotectants have been criticized for using an unrealistic time window such as pre-stroke treatment or treatment that is concurrent with stroke [42, 43]. The 4 h (delayed time point) was selected with reference to the outer limit of the therapeutic window for i.v. tPA (4.5 h from first symptom) to ensure its translational value, while the 24 h (very delayed time point) was selected to see if the therapeutic window could be extended. While it was not surprising that the 24-h (very delayed) treatment time point was ineffective, it was surprising to find that concurrent treatment was also not effective. Although the reasons for this are currently not known, we speculate that it may be related to poor uptake of the miRNA by neurons in the early phase of ischemia. Data from this study demonstrates that stroke increases the neuronal uptake of miR-20a-3p as compared to astrocytes (Fig. 5a–e), and one possibility is that it may be due to an increase in the expression of miRNA chaperone proteins. MiRNA chaperones such as the very low-density lipoprotein bind to their membrane receptors (VLDLR) to gain access to cells. Neurons, as do many other cells, increase VLDLR expression after ischemia [44]. Increasing the expression of VLDLR would allow the neuron to more efficiently internalize the microRNA. It is worth noting that VLDLR is also a predicted miR-20a-3p target. It is therefore possible that providing miR-20a-3p too early in the ischemic process reduces the expression of VLDLR resulting in insufficient uptake of the microRNA. Without sufficient uptake, miR-20a-3p is not able to be utilized efficiently and is thus not neuroprotective when given at the onset of the stroke. The 4-h (delayed) time point may be early enough to prevent massive cell death and permanent impairment, yet late enough to allow for sufficient upregulation of VLDLR, thus resulting in a unique, delayed therapeutic window.

Another unusual aspect of these studies is the beneficial effect of miR-20a-3p on both males and females after stroke. As more studies include both sexes in their experimental design, it is clear that drugs are not always equally effective in both sexes. Among the earliest drugs that displayed a sex difference in treatment outcome was the selective pan-caspase inhibitor, quinoline-Val-Asp(Ome)-CH2-O-phenoxy (Q-VD-OPh). Female mice exhibit an early release of cytochrome C and increased caspase activation after stroke, but not males, and that Q-VD-OPh treatment to females decreased infarct volume and improved neurological outcome, but this effect was not observed in male mice [45]. In contrast, PARP inhibitors were effective stroke treatments in male mice and ineffective in females, suggesting that while caspase-dependent cell death pathways are initiated in the ischemic brain of both sexes, males tend to show a preference to PARP- and NO-dependent cell death under ischemic conditions [46, 47]. Recently, we identified miR-363-3p, another stroke-protective miR, using a profiling approach which improved stroke outcomes in females but was found ineffective in males [48]. Interestingly, one of the principal targets of miR-363-3p is caspase-3, a cell death effector, which is consistent with the idea that targeting caspase pathways is therapeutic for females. Sex differences have also been reported for miR-181c, which interacts with the estrogen receptor to improve stroke recovery in females [49]. The unique aspect of the present study is that miR-20a-3p treatment is effective in middle-aged animals of both sexes, which may be linked to its predicted targets such as MMPs and mitochondrial energetics.

These results demonstrate that delayed miR-20a-3p mimic treatment improves acute stroke outcomes and identifies several key pathways by which it may be neuroprotective. However, there are several limitations to this study. Firstly, only one dose of the miRNA was tested. Previous studies from our lab have shown that a single dose of a neuroprotective miRNA is sufficient to improve stroke outcomes [48], though it is entirely possible that multiple doses may further mitigate the severity of damage after stroke. Other potential stroke therapeutics have seen success in adopting a multiple dosage schedule [50–52]. The present study provides justification for further experiments that include more frequent treatment schedules. Another limitation is that this study only assesses the therapeutic potential of miR-20a-3p in the acute phase. Depression and impaired cognitive function are significant long-term symptoms of stroke [53, 54], and improving these long-term consequences is critical for a potential stroke therapeutic. Though mitigating damage in the acute phase may be critical to improving chronic outcomes, future experiments involving chronic behavioral outcomes are necessary to more completely evaluate the therapeutic potential of miR-20a-3p. Finally, we did not assess whether or not these effects of miR-20a-3p are permanent, though there is evidence to suggest that microRNA actions are reversible [55]. Long-term studies involving multiple dosing strategies could shed light on this mechanism.

This study establishes an innovative microRNA-based therapy derived from profiling young and aging astrocytes. Astrocytes are complex cells with the ability to both promote neuronal survival and elevate neuroinflammation, implying both a protective role and a detrimental role for the astrocytes in the brain (reviewed in Ref. [56]). This study also directly compared the conditional expression of miR-20a-3p in two different cell types, establishing neurons as the primary target for miR-20a-3p–mediated neuroprotection. In view of its sex-independent effects and effective time-delayed treatment option, these studies underscore the potential of miR-20a-3p as a mediator of cell senescence and an effective therapy with translational relevance.

## Methods

### In Vivo Studies

#### Animals

All animals were purchased from Harlan Laboratories (IN). Animals were purchased as adults (6–7 months, 230–320 g) or middle aged (10–12 months, 280–360 g). All animals were maintained in a 12-h dark:12-h light cycle in AAALAC-accredited vivarium facilities. Food and water were available ad libitum. A week after arrival, females were smeared daily for 14–21 days to determine estrous status (Jezierski and Sohrabji 2001). Vaginal cells were collected using cotton swabs and placed on slides, and cytology was examined at a low magnification. Adult females with a normal estrous cycle of 4–6 days were included in the study. Middle-aged females were included if cytology indicated they were in constant diestrus for at least 7 consecutive days. Adult animals and middle-aged animals were at an average of 7 months and 11.5 months, respectively, at the time of middle cerebral artery occlusion (MCAo). Within each age and sex, animals were assigned randomly to the treatment groups. A total of 143 animals were used in these studies with group sizes of 5–12. All experimental procedures were conducted in accordance with ARRIVE guidelines [57].

### Surgical Procedures

#### AAV5 Injections

Animals were anesthetized (ketamine: 87 mg/kg; xylazine: 13 mg/kg) and placed in a stereotaxic instrument for delivery of the adenovirus construct to the cortex and striatum or the striatum alone. The construct was designed as follows: Recombinant adeno-associated virus serotype 5 (AAV5) was packaged (SignaGen, MD) with the miR-20a-3p gene downstream of the astrocyte-specific reporter GFAP and a tetracycline-inducible element (TetOn) and tagged with the mCherry reporter gene. The control construct consisted of an identical shuttle vector without the miR-20a-3p gene. In preliminary studies, the construct was injected at two doses (2.5 × 10^11^ VP/ml and 2.5 × 10^10^ VP/ml) and delivered stereotaxically either to the striatum as a single injection or to the cortex and striatum as two injections. The following coordinates were used relative to bregma on the left hemisphere: for the striatum: + 0.9 mm anterior/posterior, + 3.6 mm medial/lateral, and − 6.5 mm relative to the dura, and for the cortex: + 0.9 mm anterior/posterior, + 5.5 mm medial/lateral, and − 6.0 mm relative to the dura. Coordinates for the single striatal injections are as follows: + 0.9 mm anterior/posterior, + 2.8 mm medial/lateral, and − 6.5 mm relative to the dura. In each case, a needle attached to a Hamilton syringe was lowered to the appropriate depth and rAAV-TetOn-GFAP-miR-20a-3p-mCherry was delivered slowly into the parenchyma at a rate of 0.5 µl/min for a total of 3.5 µl. Animals were allowed to recover for 6 weeks after injections to allow full integration of the viral particles prior to MCAo.

#### Middle Cerebral Artery Occlusion

All animals were subjected to stereotaxic surgery to occlude the left middle cerebral artery as reported in Refs. [8, 14, 58]. Briefly, MCA occlusion was induced by stereotaxic microinjection of endothelin-1 (3 µl, 1:2 dilution in DPBS of 1 mg/ml endothelin-1 stock; American Peptide Company, Inc., CA). ET-1 was injected adjacent to the middle cerebral artery at the following coordinates relative to bregma: + 0.9 mm anterior/posterior, + 3.4 mm medial/lateral, and − 8.5 mm relative to the dura. For micronome assays, animals were terminated 2 days post stroke. The same procedure was followed for sham surgeries without the injection of endothelin-1. For miR mimic treatment experiments, animals were administered tail vein injections either immediately after stroke (IT), 4 h post stroke (DT), or 24 h post stroke (VDT) with 300 µl (7 µg/kg) of either miRNA mimic or negative control: miR-20a-3p (ACUGCAUUACGAGCACUUACA) oligonucleotide sequence (Thermo Fisher, Grand Island, NY) in In Vivo RNA-LANCEr II (Bioo Scientific, Austin, TX). For rAAV experiments, animals were injected i.p. with doxycycline, a stable Tet analogue, to activate the construct. Animals were terminated at 5 days post MCAo for these experiments. At termination, the brain was rapidly removed and processed for TTC staining to assess infarct volume or was collected for RNA and protein extraction. For FAM-miR-20a-3p experiments, animals were injected with a FAM-labeled miR-20a-3p mimic 4 h after MCAo or sham surgery. These animals were perfused at 48 h post MCAo. The brains were then extracted and fixed, sucrose loaded, cryosectioned at 30 µm per slice, and then mounted on glass slides. The slides were then incubated with the appropriate primary and secondary antibodies (see “Immunohistochemistry”) and imaged using a FV12-IX83 confocal microscope. The images were then quantified using the following methods: 200 cells (identified by the nuclear dye DAPI) that demonstrated co-localization with the FAM-miR-20a-3p oligo were randomly selected, and the proportion of those cells that were NeuN+ or GFAP+ was quantified.

##### Infarct Volume

Infarct volume estimation was performed on animals terminated on day 5 post stroke using our previously described procedures [59]. Briefly, brain slices (2 mm thick) between − 2.00 and + 4.00 mm from bregma were incubated in a 2% TTC solution at 37 °C for 20 min and then photographed using a Nikon E950 digital camera attached to a dissecting microscope. Digitized images were coded and analyzed by an investigator blind to the code. Infarct volume was determined using the Quantity One software package (Bio-Rad, CA) or ImageJ (NIH). Hemorrhagic loci were also visualized in TTC-stained sections by an investigator blind to the code.

#### Behavioral Assays

##### Adhesive Removal Task

Sensory motor performance was assessed using procedures described previously for the adhesive removal test [48, 60]. Briefly, two pieces of adhesive-backed foam tape (1 in. × 0.5 in.) were used as bilateral tactile stimuli attached to the palmar surface of the paw of each forelimb. For each forelimb, the time it took to remove each stimulus (tape) from the forelimbs was recorded during three trials per day for each forepaw. Animals were allowed to rest for 5 min between sessions, and each test session had a maximum time limit of 120 s.

##### Vibrissae-Evoked Forelimb Placement Task

Stroke injury was assessed using the vibrissae-evoked forelimb placement task, which was performed pre and post MCAo (described by [59, 61]). Briefly, animals were subjected to same-side placing trials and cross-midline placing trials elicited by stimulating ipsilesional and contralesional vibrissae. Vibrissae-evoked forelimb placement trials revealed a significant loss of right paw placement in all animals post stroke, indicating left-sided cortical-striatal infarction (Supplemental Fig. 4).

##### Astrocyte microRNA

Astrocytes were harvested from the ischemic hemisphere 2 days post stroke, using procedures published in Ref. [23]. Briefly, tissues were dissociated using a neural dissociation kit (trypsin) and cells were passed through a 30-µm filter to obtain a single cell suspension. Following myelin removal, cells were collected by positive selection using anti-GLAST antibody (1:5) for 10 min. GLAST was selected as a marker because it is an astrocyte-specific, membrane-associated protein, and previous work has established that astrocytes harvested 48 h after ischemia express glutamate transporters (GLT-1 and GLAST) and display no age differences in expression.

##### miRnome Profiling

Astrocyte miRnome was assessed in brain astrocytes from the ischemic hemispheres (*n* = 6 in each experimental group). Two panels covering 752 mouse and rat miRNAs were used (miRCURY LNA miRNA miRNome PCR Panels).

##### RNA Extraction

RNA was extracted from serum and astrocytes using the miRNeasy Kit (Qiagen, CA) following the manufacturer’s instructions, as described in Ref. [14]. Sample purity was assessed by NanoDrop technology, and a ratio of 1.8 was considered acceptable. Samples were stored at − 20 °C until use.

##### PCR Amplification

Template RNA (25 ng total RNA per sample) was incubated with reverse transcriptase for 60 min at 42 °C, followed by heat inactivation of the enzyme (5 min at 95 °C) and used immediately. cDNA was diluted 80-fold and then incubated with SYBR® Green master mix. Ten microliters was dispensed to each tube. An activation/denaturation step (95 °C, 10 min) precedes 40 amplification cycles each at 95 °C for 10 s and at 60 °C for 1 min, with a ramp rate of 1.6 °C/s. MiRNA primers were LNA modified (Exiqon, Woburn, MA) which allows for uniform T*m* and confers greater specificity. Samples were then subject to PCR amplification of U6 as a housekeeping gene. Delta CT values of miR-20a-3p were obtained by subtracting the U6 value, and ddCT values were obtained by subtracting the mean dCT of the adult female group from each value in all groups. The fold change was expressed as the inverse log of ddCT(1/2^ddCT^).

MiRNA expression data obtained from miRnome panels were uploaded into the GSEA (GeneSifter® Analysis Edition) software program (Geospiza). Differences in miRNA expression were identified using a two-way ANOVA using age and sex as two independent factors, with Benjamini and Hochberg correction for false discovery rate for multiple comparisons at a cutoff (*α*) of 0.05.

### Protein Analysis

#### Immunohistochemistry

Immunofluorescence for NeuN, ALDH1L1, and GFAP were performed on 30-µm brain sections mounted on glass slides. The sections were incubated with a blocking buffer (5% bovine serum albumin, 0.1% Triton X-100 in PBS, pH 7.4) for 1 h at room temperature. Sections were then incubated overnight at 4 °C with primary antibody (NeuN: anti-mouse [EMD Millipore], 1:250 µl; GFAP: anti-rabbit [Sigma-Aldrich], 1:3000 µl; Iba1: anti-rabbit [Wako Chemicals], 1:500 µl). Secondary antibodies (Alexa Fluor 488 and 594 anti-rabbit and anti-mouse) were then used at a 1:500 µl dilution at room temperature for 1 h. The sections were then washed thrice with PBS and then cover slipped with mounting media containing the nuclear dye DAPI (Fluoroshield, Abcam). Sections were visualized and imaged using an FV12-IX83 confocal microscope.

#### Protein Extraction

Cell proteins from the ischemic hemisphere (cortex and striatum) from animals terminated at 48 h were harvested and lysed in RIPA lysis buffer (Thermo Scientific, Grand Island, NY) and centrifuged at 20,000 rpm for 30 min. Supernatant was collected and stored at − 20 °C until further analysis. Protein concentrations were determined using the BCA protein assay kit (Pierce, Rockford, IL).

#### Western Blots

Protein extract (30 µg) from the ischemic hemisphere was loaded into 4–12% Novex gels and run at 60 V for 30 min and then at 100 V for 90 min. The protein was then transferred onto a PDVF membrane and probed with an antibody for Drp1 (anti-mouse, Abcam, 1:1000 µl) and conjugated to a fluorescent secondary antibody (LiCOR goat anti-mouse IRDye 680RD), normalizing to total protein (LiCOR Total Protein Stain).

#### Zymography

Protein extract (25 µg) from the ischemic hemisphere of middle-aged female rats post MCAo was loaded into a 10% Novex Zymogram Plus (gelatin) gel and run at 60 V for 30 min and then at 100 V for 90 min in a non-reducing sample buffer. The gel was then washed and incubated for 24 h at 37 °C. The gel was then stained with Coomassie blue for 30 min and then destained until clear bands could be seen.

#### IL-17a Assay

IL-17A expression was measured using the Rat IL-17A Platinum ELISA (Thermo Scientific, Grand Island, NY) according to the manufacturer’s instructions. Briefly, standards, controls, and aliquots of serum and protein lysates from ischemic cortex and striatum samples were loaded into a 96-well plate pre-coated with antibodies specific for IL-17A and followed by the addition of 100 µl of biotin-conjugated anti-rat IL-17A antibody and incubated at room temperature for 2 h on an orbital microplate shaker at 400 rpm for 30 s. With intervening washes, plates were sequentially incubated with 100 µl of streptavidin-HRP for 2 h, and 100 µl of TMB substrate solution for 30 min. The color reaction was stopped by an equal volume of stop solution and read at 450 nm in a microplate reader (Tecan, Switzerland). Standard curves were established from optical densities of wells containing known dilutions of the standard (1.6–100 pg/ml), and sample measurements were interpolated from standard curves. The experiments were performed in duplicates.

### In Vitro Studies

#### Cell Culture and OGD

Human astrocytes and neurons from male and female donors of 18–20 gestational weeks of age were purchased from ScienCell Research Laboratories, grown in “Neural Growth Media (NGM)”, consisting of Neurobasal media with 2% B-27 supplement, 2% heat-inactivated gelded horse serum, 1% GlutaMAX, 1% penicillin, 0.1% ascorbic acid, 0.05% ampicillin, and 0.05% kanamycin, and plated in T-25 or T-75 cell culture-treated flasks. For experiments, cells were plated in poly-d-lysine—coated 6- or 96-well plates or in glass-bottom culture dishes in densities appropriate for the assay, which were ascertained via cell titration. Cultures were grown in normoxic conditions (5% CO_2_ and 21% O_2_, 37 °C) until confluent. Astrocytes were then subject to OGD (1% O_2_, 95% N_2_, and 5% CO_2_ in glucose-free DMEM, 37 °C) for 6 h with miR-20a-3p mimic (50 nM), scrambled miRNA (50 nM), or vehicle (PBS). Neurons were subjected to the same OGD conditions for 30 min. Culture media was collected, and cells were used for assays or processed for RNA isolation.

#### Calcein Assay

Cells were seeded at a density of 2 × 10^4^ in a 96-well plate and subjected to OGD and treatment conditions for 6 h. Cell viability was determined using the Calcein-AM dye (Life Technologies, CA). After OGD, cells were incubated with Calcein-AM (2.5 µm) in Calcein-AM buffer for 20 min at 37 °C, and fluorescence was measured on a plate reader (Tecan, Switzerland) with excitation/emission set at 480 nm and 530 nm, respectively.

#### FRAP Analysis

Cells were seeded at a density of 1 × 10^5^ into 2-ml glass-bottom culture dishes, cultured for 2 days, and then subjected to OGD and treatment conditions for 6 h. Culture media was collected, and cells were washed twice with gas-free recording buffer. Cells were then incubated with 120 nM MitoTracker Deep Red in gas-free recording buffer (154 mM NaCl, 5 mM KCl, 2 mM CaCl_2_·H_2_O, 0.5 mM MgCl_2_·H_2_O, 5 mM d-glucose, 10 mM HEPES) for 1 h. After incubation, cells were washed twice with gas-free recording buffer and imaged using confocal microscopy (FV12-IX83). Individual cells with MitoTracker Deep Red labeling were identified, and a region of interest containing a dense area of mitochondria close to the soma was selected. Pre-activation was recorded for 30 s, cells were bleached with high-intensity laser (405 nm, 80% power of total laser output) for 3 s, and subsequent activity was recorded for 90 s post bleach. Fluorescence intensity over time was plotted, and cells that did not achieve at least 75% bleaching were excluded from analysis.

#### Mitochondrial Respiration

Cells were seeded at a density of 4 × 10^4^ in 96-well Seahorse microplates 15 h before OGD. After OGD, cells were washed twice with Seahorse XF DMEM medium (pH 7.4) and incubated for 45 min in a CO_2_-free incubator. The Mito Stress Test was then performed according to the manufacturer’s instructions. Briefly, this includes taking basal oxygen consumption measurements and then oxygen consumption measurements on the Seahorse XFe96 Analyzer after serial injections of oligomycin, FCCP, and rotenone/antimycin A.

### Statistical Analysis

Power analysis for group sizes was computed based on effect sizes seen in previous data and pilot studies. In order to achieve a power of 0.9 (1 − *β*) and type 1 error rate (*α* = 0.05), the minimum sample size is 5. For these studies, group sizes ranged from 5 to 10. For comparisons between adult and middle-aged males and females on miR-20a-3p expression, a two-way ANOVA with post hoc comparisons was performed. For behavioral tests, a two-way ANOVA coded for repeated measure was used for each group, comparing the values obtained pre and post stroke. For all other tests, an unpaired *t* test was performed. Group differences were considered significant at *p* < 0.05 in each case. All in vitro assays were conducted with 3–5 replicate runs, and each run consisted of 3–12 technical replicates. Statistical analysis was performed using unpaired *t* tests for the Calcein assay, multiple *t* tests (one per time point) for FRAP assay, and 2-way ANOVA with post hoc comparisons for Seahorse assay. All statistical analyses were performed using GraphPad Prism software (v. 9.0).

## Supplementary Information

Below is the link to the electronic supplementary material.

Supplementary file1 (TIFF 8792 kb)
Supplemental Fig. 1 Analysis of predicted miR-20a-3p targets. In silico analysis was performed on predicted gene targets of miR-20a-3p using the TargetScan and miRWalk database. Graph shows significant Gene Ontology pathways and the percentage of predicted miR-20a-3p target genes in each GO pathway.

Supplementary file2 (TIFF 8792 kb)
Supplemental Fig. 2 GFAP Immunohistochemistry of in vitro human cells. Representative image of human astrocytes (A) and human neurons (B) probed for antibodies to GFAP or NeuN, respectively (green). Control cultures incubated with (top right of panel) secondary antibody only (no primary) or (bottom right of panel) primary antibody only (no secondary). In all cases, cells were counter stained with the nuclear dye DAPI (blue).

Supplementary file3 (TIFF 8792 kb)
Supplemental Fig. 3 Characterization of astrocyte-specific viral vector for conditional expression of miR-20a-3p: (A) Schematic representation of the viral construct rAAV5 containing the miR-20a-3p gene downstream of the GFAP/Tet inducible promoter and linked to an mCherry reporter. (B) Schematic of the control vector. (C) Immunohistochemistry for GFAP, NeuN, and Iba1 on sections from rats injected with the rAAV construct and treated either with Dox (upper panel) or vehicle (lower panel). Arrows indicate mCherry localized to GFAP immunopositive cells.

Supplementary file4 (TIFF 8792 kb)
Supplemental Fig. 4 Characterization of neuron-specific viral vector for conditional expression of miR-20a-3p: (A) Schematic representation of the viral construct rAAV5 containing the miR-20a-3p gene downstream of the NSE/Tet inducible promoter and linked to an mCherry reporter. (B) Schematic of the control vector. (C) Immunohistochemistry for GFAP, NeuN, and Iba1 on sections from rats injected with the rAAV construct and treated either with Dox (upper panel) or vehicle (lower panel). Arrows indicate mCherry localized to NeuN immunopositive cells.

Supplementary file5 (TIFF 8792 kb)
Supplemental Fig. 5 Effect of intravenous miR20a-3p mimics treatment on stroke outcomes in middle-aged males: Middle-aged male rats were injected with miR-20a-3p mimics or scrambled oligos 4 h after MCAo. (A) Representative TTC-stained coronal sections from scrambled oligo and miR-20a-3p injected animals. (B) Histogram depicts average infarct volume (±SEM) normalized to the volume of the non-ischemic hemisphere. (C) Sensory motor performance on the adhesive removal test was evaluated before and after stroke. Histograms depict mean (±SEM) latency in seconds to remove the tape. *p ≤ 0.05. Middle-aged male N = 6 (control) and 5 (treatment).

Supplementary file6 (TIFF 8792 kb)
Supplemental Fig. 6 Vibrissae-evoked forelimb placement task: The vibrissae-evoked forelimb placement task was performed on all in vivo experiments: (A) GFAP rAAV animals. (B) NSE rAAV animals. (C) Delayed treatment. (D) Immediate treatment. (E) Very delayed treatment. For all groups, there is an effect of time on task performance. **p ≤ 0.01, ***p ≤ 0.001, ****p ≤ 0.0001.

## Data Availability

This study includes no data deposited in external repositories. Requests for resources and reagents should be directed to the Corresponding Author, Farida Sohrabji (f-sohrabji@tamu.edu).
