# Supplementary figures and images for: Functional Assessment of Stroke-Induced Regulation of miR-20a-3p and Its Role as a Neuroprotectant
Source: Transl Stroke Res. 2021 Sep 27;13(3):432–48. doi: 10.1007/s12975-021-00945-x (PMC9046320; doi:10.1007/s12975-021-00945-x)

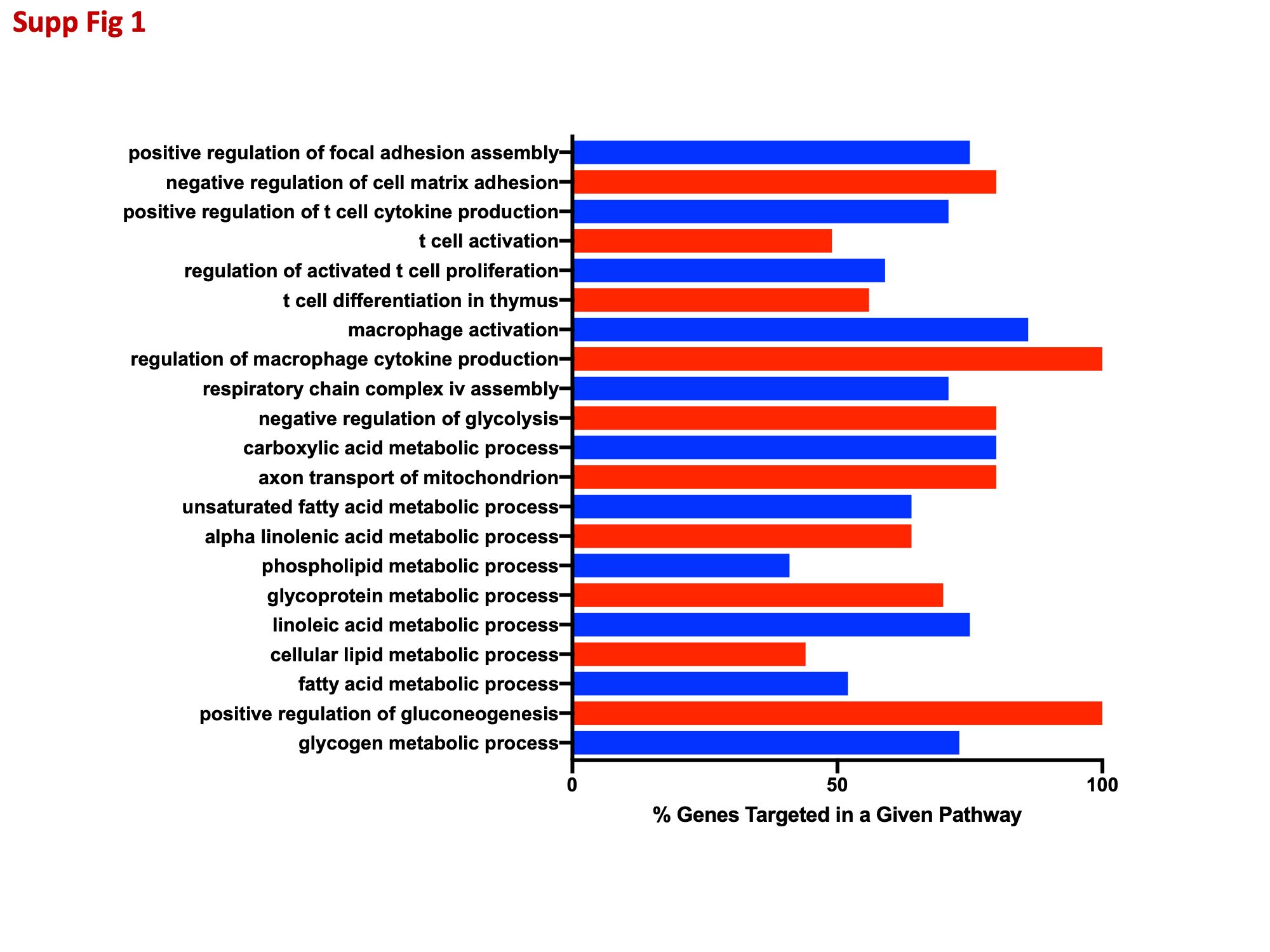

Supplement: Supplementary file 1 — Supplementary file1 (TIFF 8792 kb) Supplemental Fig. 1 Analysis of predicted miR-20a-3p targets. In silico analysis was performed on predicted gene targets of miR-20a-3p using the TargetScan and miRWalk database. Graph shows significant Gene Ontology pathways and the percentage of predicted miR-20a-3p target genes in each GO pathway. [file 12975_2021_945_MOESM1_ESM.tiff]

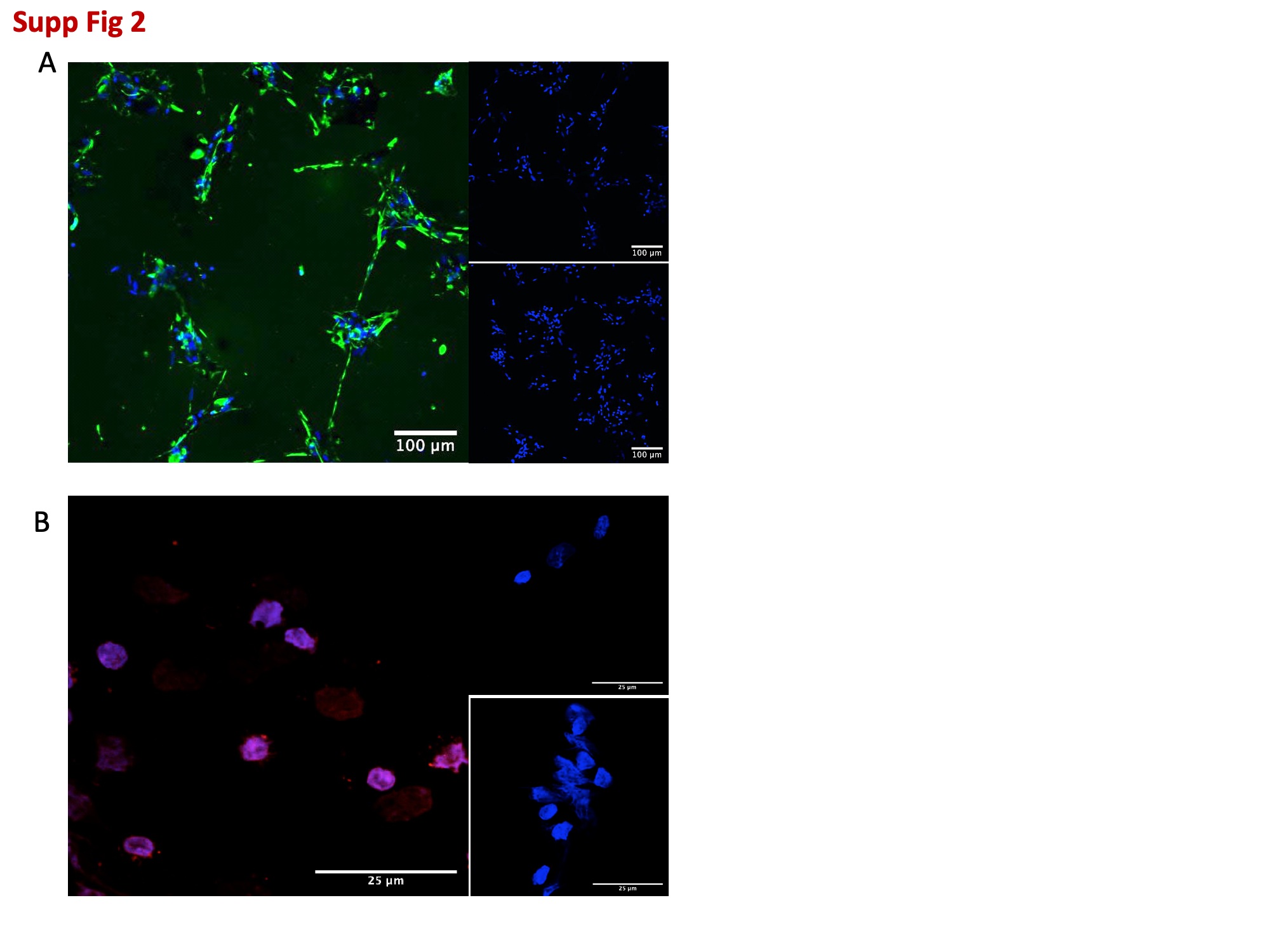

Supplement: Supplementary file 2 — Supplementary file2 (TIFF 8792 kb) Supplemental Fig. 2 GFAP Immunohistochemistry of in vitro human cells. Representative image of human astrocytes (A) and human neurons (B) probed for antibodies to GFAP or NeuN, respectively (green). Control cultures incubated with (top right of panel) secondary antibody only (no primary) or (bottom right of panel) primary antibody only (no secondary). In all cases, cells were counter stained with the nuclear dye DAPI (blue). [file 12975_2021_945_MOESM2_ESM.tiff]

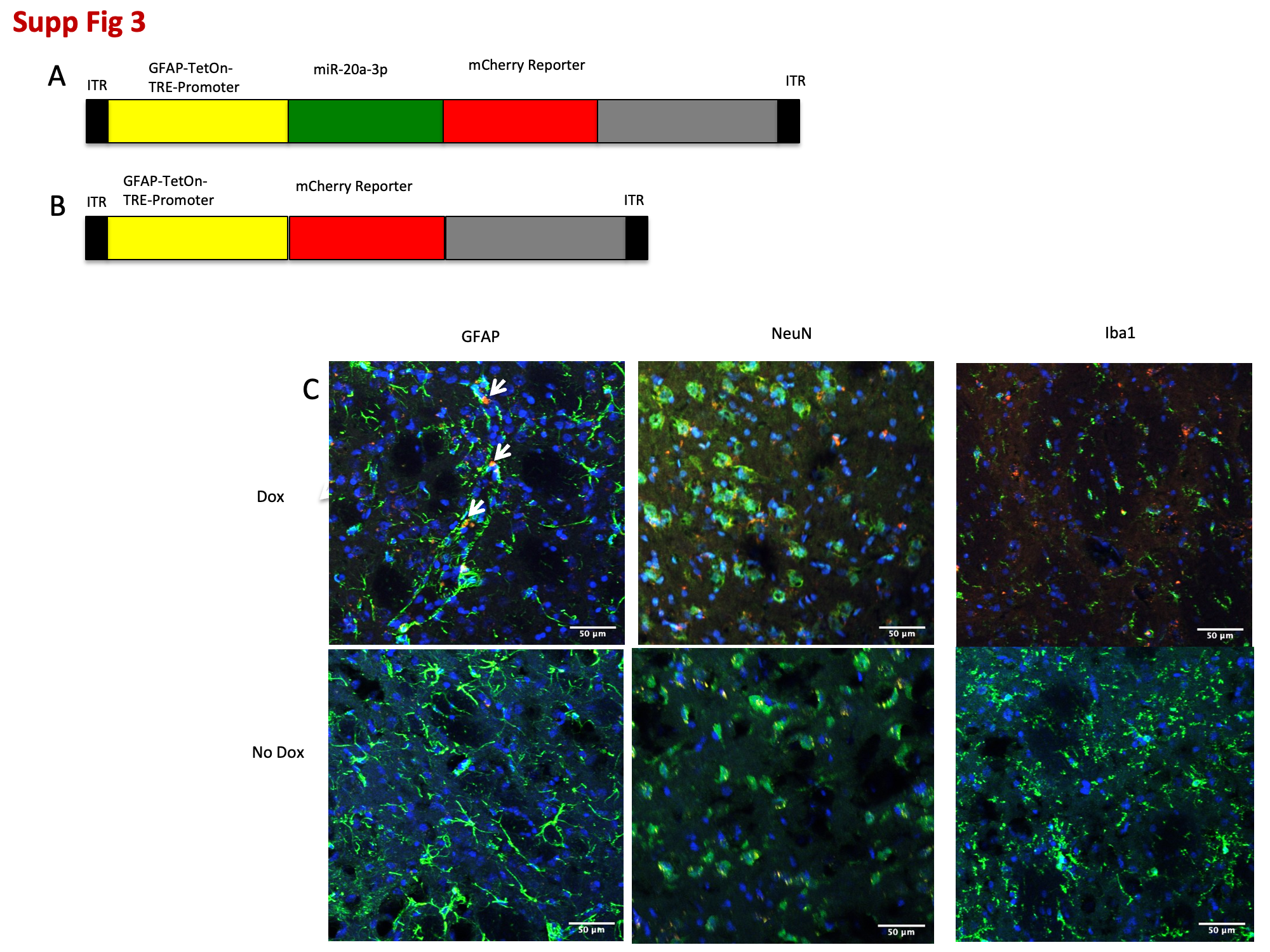

Supplement: Supplementary file 3 — Supplementary file3 (TIFF 8792 kb) Supplemental Fig. 3 Characterization of astrocyte-specific viral vector for conditional expression of miR-20a-3p: (A) Schematic representation of the viral construct rAAV5 containing the miR-20a-3p gene downstream of the GFAP/Tet inducible promoter and linked to an mCherry reporter. (B) Schematic of the control vector. (C) Immunohistochemistry for GFAP, NeuN, and Iba1 on sections from rats injected with the rAAV construct and treated either with Dox (upper panel) or vehicle (lower panel). Arrows indicate mCherry localized to GFAP immunopositive cells. [file 12975_2021_945_MOESM3_ESM.tiff]

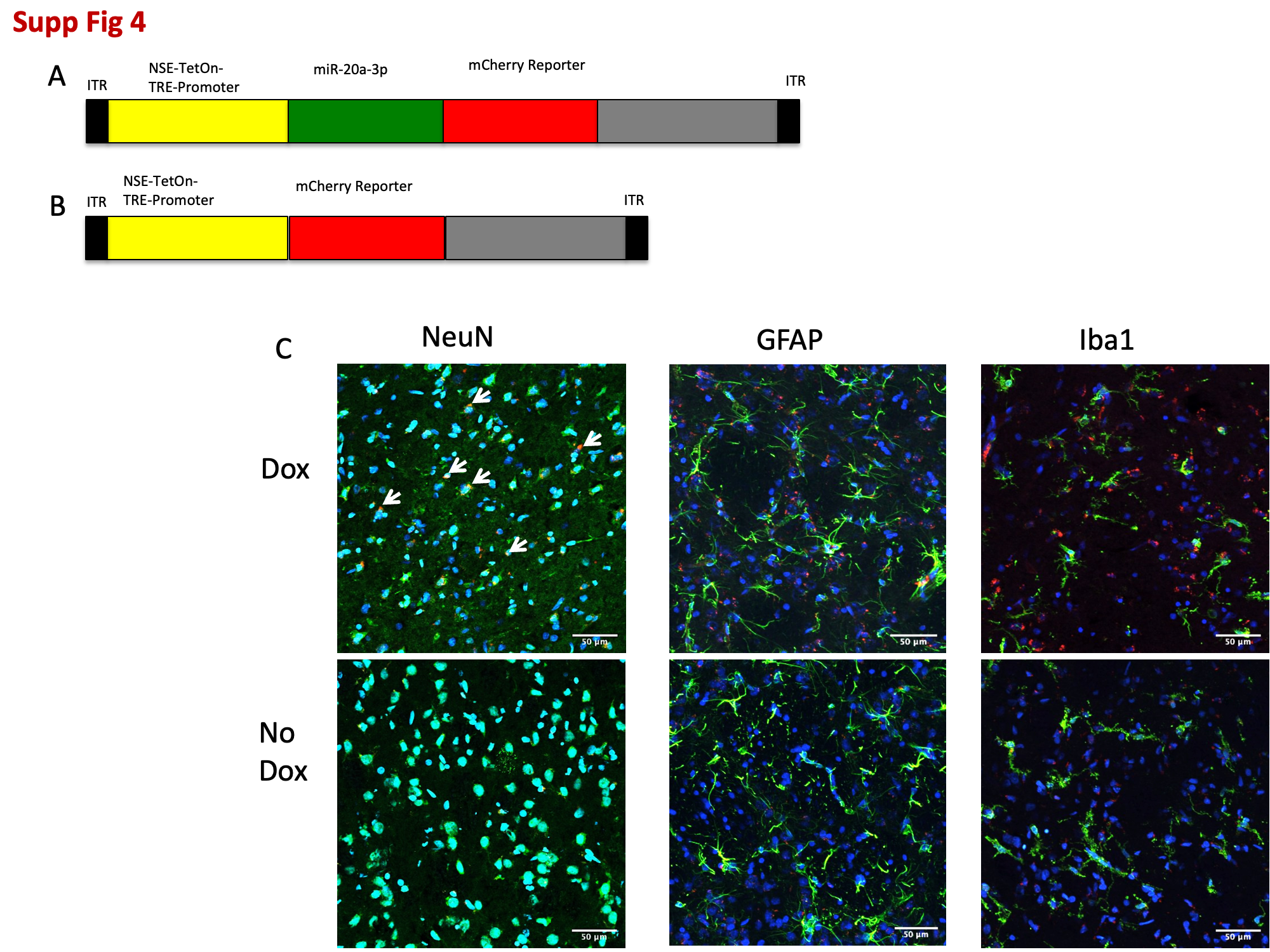

Supplement: Supplementary file 4 — Supplementary file4 (TIFF 8792 kb) Supplemental Fig. 4 Characterization of neuron-specific viral vector for conditional expression of miR-20a-3p: (A) Schematic representation of the viral construct rAAV5 containing the miR-20a-3p gene downstream of the NSE/Tet inducible promoter and linked to an mCherry reporter. (B) Schematic of the control vector. (C) Immunohistochemistry for GFAP, NeuN, and Iba1 on sections from rats injected with the rAAV construct and treated either with Dox (upper panel) or vehicle (lower panel). Arrows indicate mCherry localized to NeuN immunopositive cells. [file 12975_2021_945_MOESM4_ESM.tiff]

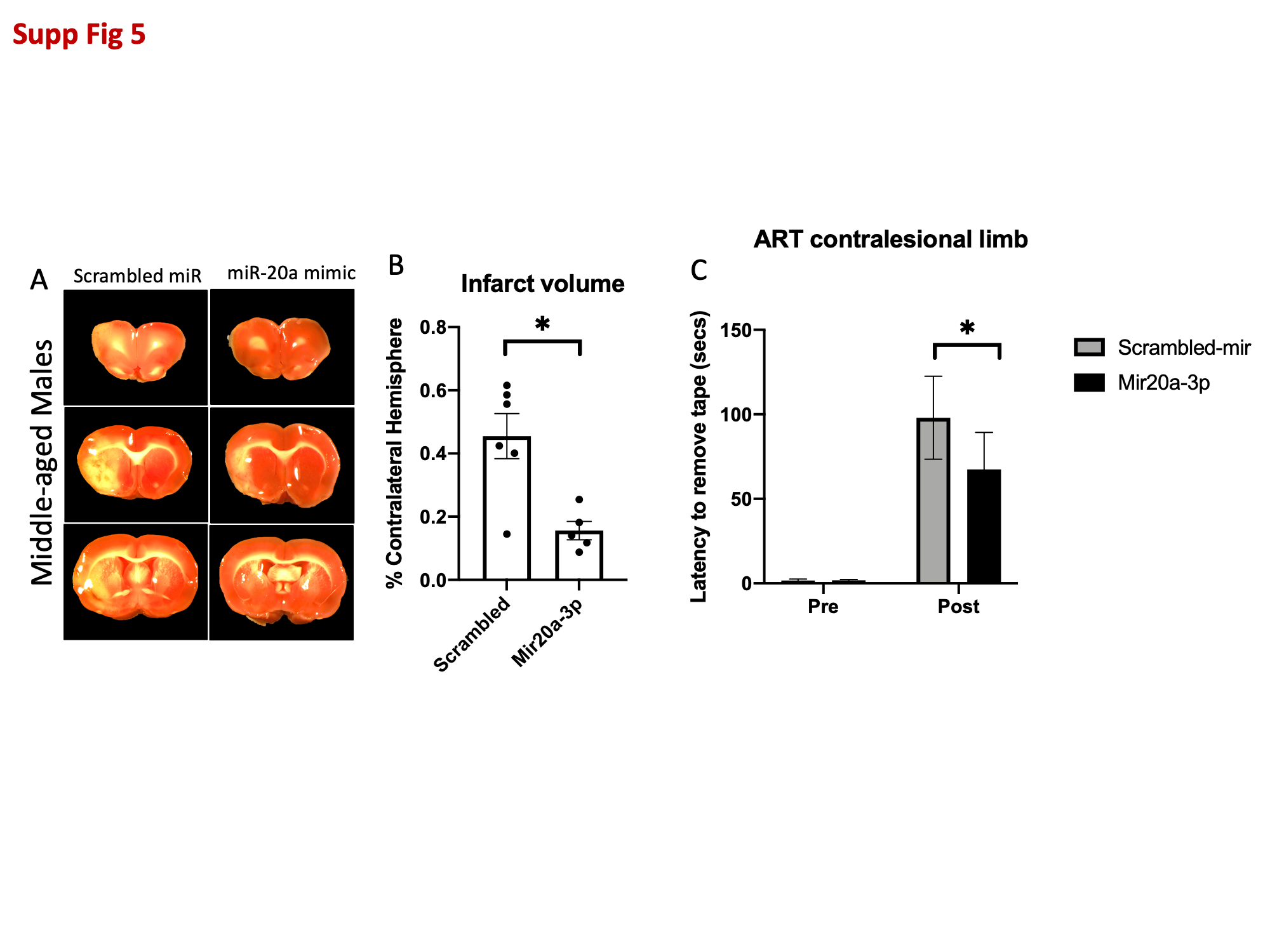

Supplement: Supplementary file 5 — Supplementary file5 (TIFF 8792 kb) Supplemental Fig. 5 Effect of intravenous miR20a-3p mimics treatment on stroke outcomes in middle-aged males: Middle-aged male rats were injected with miR-20a-3p mimics or scrambled oligos 4 h after MCAo. (A) Representative TTC-stained coronal sections from scrambled oligo and miR-20a-3p injected animals. (B) Histogram depicts average infarct volume (±SEM) normalized to the volume of the non-ischemic hemisphere. (C) Sensory motor performance on the adhesive removal test was evaluated before and after stroke. Histograms depict mean (±SEM) latency in seconds to remove the tape. *p ≤ 0.05. Middle-aged male N = 6 (control) and 5 (treatment). [file 12975_2021_945_MOESM5_ESM.tiff]

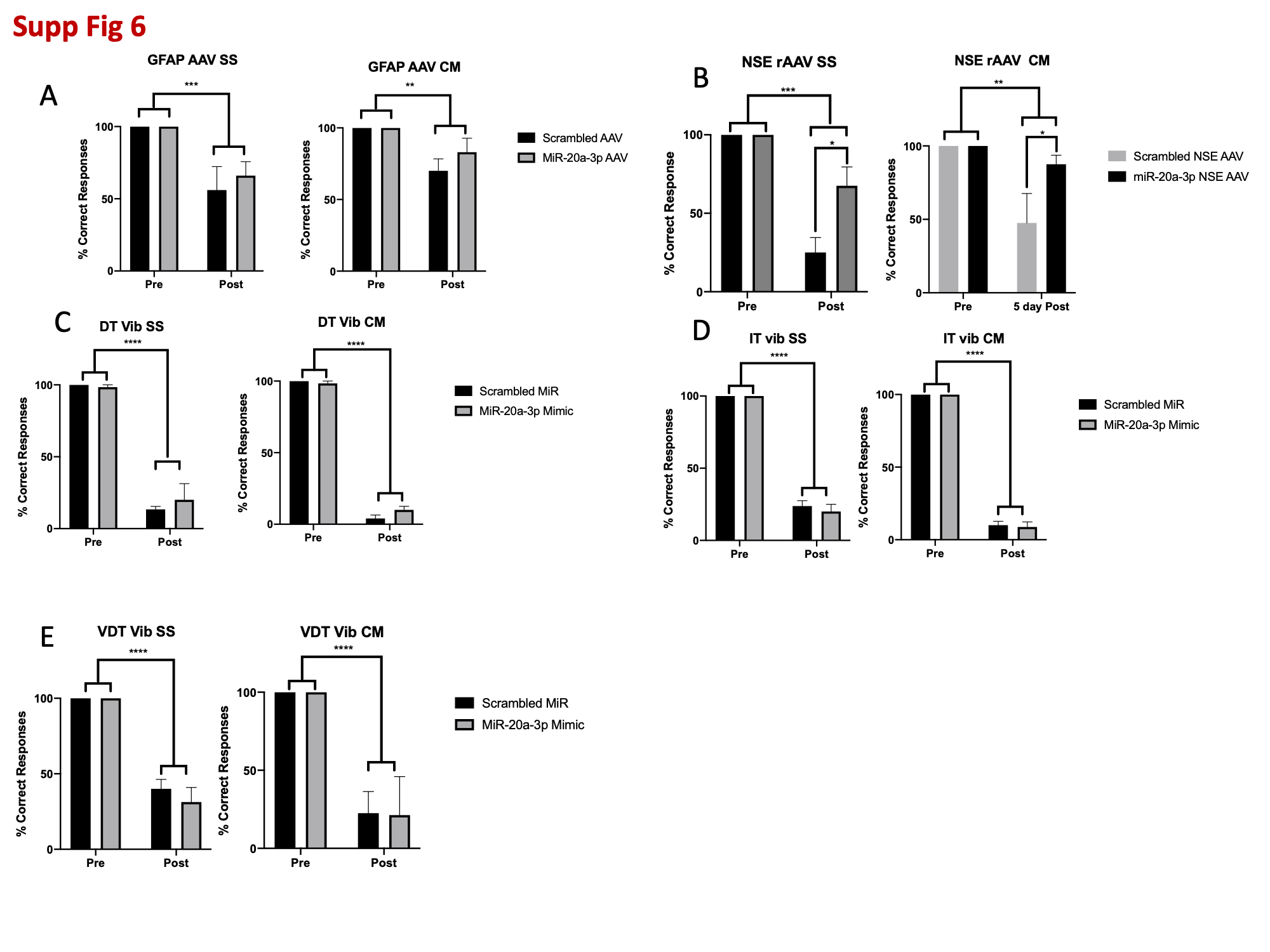

Supplement: Supplementary file 6 — Supplementary file6 (TIFF 8792 kb) Supplemental Fig. 6 Vibrissae-evoked forelimb placement task: The vibrissae-evoked forelimb placement task was performed on all in vivo experiments: (A) GFAP rAAV animals. (B) NSE rAAV animals. (C) Delayed treatment. (D) Immediate treatment. (E) Very delayed treatment. For all groups, there is an effect of time on task performance. **p ≤ 0.01, ***p ≤ 0.001, ****p ≤ 0.0001. [file 12975_2021_945_MOESM6_ESM.tiff]
